# Machine-learning classification using neuroimaging data in schizophrenia, autism, ultra-high risk and first-episode psychosis

**DOI:** 10.1038/s41398-020-00965-5

**Published:** 2020-08-17

**Authors:** Walid Yassin, Hironori Nakatani, Yinghan Zhu, Masaki Kojima, Keiho Owada, Hitoshi Kuwabara, Wataru Gonoi, Yuta Aoki, Hidemasa Takao, Tatsunobu Natsubori, Norichika Iwashiro, Kiyoto Kasai, Yukiko Kano, Osamu Abe, Hidenori Yamasue, Shinsuke Koike

**Affiliations:** 1 Department of Child Neuropsychiatry, Graduate School of Medicine, The University of Tokyo, Tokyo, 113-8655 Japan; 2 Department of Information Media Technology, School of Information and Telecommunication Engineering, Tokai University, Tokyo, 108-8619 Japan; 3 Center for Evolutionary Cognitive Sciences, Graduate School of Arts and Sciences, The University of Tokyo, Tokyo, 153-8902 Japan; 4 Department of Psychiatry, Hamamatsu University School of Medicine, Hamamatsu City, 431-3192 Japan; 5 Department of Radiology, Graduate School of Medicine, The University of Tokyo, Tokyo, 113-8655 Japan; 6 Medical Institute of Developmental Disabilities Research, Showa University, Tokyo, Japan; 7 Department of Neuropsychiatry, Graduate School of Medicine, The University of Tokyo, Tokyo, 113-8655 Japan; 8 International Research Center for Neurointelligence (WPI-IRCN), UTIAS, The University of Tokyo, 7-3-1 Hongo, Bunkyo-ku Tokyo, 113-8654 Japan; 9 University of Tokyo Institute for Diversity & Adaptation of Human Mind (UTIDAHM), Tokyo, 153-8902 Japan; 10 Center for Integrative Science of Human Behavior, Graduate School of Arts and Sciences, The University of Tokyo, 3-8-1 Komaba, Meguro-ku, Tokyo, 153-8902 Japan

**Keywords:** Diagnostic markers, Predictive markers, Prognostic markers, Autism spectrum disorders, Schizophrenia

## Abstract

Neuropsychiatric disorders are diagnosed based on behavioral criteria, which makes the diagnosis challenging. Objective biomarkers such as neuroimaging are needed, and when coupled with machine learning, can assist the diagnostic decision and increase its reliability. Sixty-four schizophrenia, 36 autism spectrum disorder (ASD), and 106 typically developing individuals were analyzed. FreeSurfer was used to obtain the data from the participant’s brain scans. Six classifiers were utilized to classify the subjects. Subsequently, 26 ultra-high risk for psychosis (UHR) and 17 first-episode psychosis (FEP) subjects were run through the trained classifiers. Lastly, the classifiers’ output of the patient groups was correlated with their clinical severity. All six classifiers performed relatively well to distinguish the subject groups, especially support vector machine (SVM) and Logistic regression (LR). Cortical thickness and subcortical volume feature groups were most useful for the classification. LR and SVM were highly consistent with clinical indices of ASD. When UHR and FEP groups were run with the trained classifiers, majority of the cases were classified as schizophrenia, none as ASD. Overall, SVM and LR were the best performing classifiers. Cortical thickness and subcortical volume were most useful for the classification, compared to surface area. LR, SVM, and DT’s output were clinically informative. The trained classifiers were able to help predict the diagnostic category of both UHR and FEP Individuals.

## Introduction

The current diagnostic model in psychiatry, while the best available, is not highly reliable due to three main factors: patient heterogeneity (i.e., patient’s psychological state, their ability to provide reliable information, and differences in clinical presentation), clinician inconsistency (i.e., different opinions on the same case) and nomenclature inadequacy^1,2^. As nosology is a key aspect of psychiatry, on which patient assessment and treatment options are based, it would be helpful to have a layer of appraisal centered around objective evaluations to establish a more reliable classification decision. Neuroimaging is one objective measure that might facilitate the diagnostic process, yet it is not currently used in aiding the diagnostic decision in psychiatry, despite much interest.

Machine learning uses statistical methods to find patterns in large amount of data. The learning process starts with the data at hand and improves autonomously over time. Recent advances in machine learning, combined with neuroimaging techniques, are capable of assessing differences in local morphological features of various brain subregions to elucidate novel disorder-related brain patterns^3–5^. Such patterns can be used by computational models to build classifiers for the purpose of aiding the diagnostic decision. Several studies were conducted using similar models to classify patients into their respective diagnostic category for both autism spectrum disorder (ASD)^6–10^ and schizophrenia^11–16^. However, the majority of these studies focused on distinguishing between typically developing (TD) individuals and those with psychiatric disorders^4,11,17^. This classification is important for identifying brain patterns that are different from what is considered typical, but does not inform about the variations between different patient groups which is essential for a reliable psychiatric nosology and understanding the overlap between different neuropsychiatric disorders^18^. Using both approaches, on the other hand, provides a clearer picture about the nature of the disorders and how they differ from one another. Moreover, investigating how the classifiers that are pretrained on distinct clinical phenotypes would perform on “intermediate phenotypes” or early disease states, aids in the quantification of disease progression, predicting outcome, and understanding the intersection between different nosological, phenotypic and neurobiological continua.

Classifying psychiatric disorders, especially schizophrenia and ASD, has been performed using different neuroimaging modalities. In structural magnetic resonance imaging (MRI), several studies classified patients based on a single modality, such as voxel based morphometry^19,20^, cortical thickness^3,4^, or surface morphological measures^21^. Moreover, most of these studies use a single classifier to perform the classification^15,22–27^. Few have conducted the analysis using multiple classifiers, which is important to avoid bias towards a particular classifier^28^. To our knowledge, there is no study yet that has compared several brain indices, such as cortical thickness, surface area, and subcortical volume using multiple machine learning algorithms between schizophrenia and ASD, and assessed the performance of these classifiers on at-risk and early disease stage patients.

Thus, the current study was aimed to (i) construct and compare classifiers that can distinguish between individuals with schizophrenia, ASD, and TD based on their MRI scans, (ii) uncover the most important brain feature groups contributing to the classification, (iii) assess the consistency of the classifiers with clinical severity, and (iv) predict the diagnostic category of ultra-high risk for psychosis (UHR) and first-episode psychosis (FEP) subjects using the classifiers pretrained on a combination of ASD, TD, and/or schizophrenia data.

## Methods

### Participants

The data of 131 schizophrenia spectrum (26 UHR, 25 FEP, and 80 schizophrenia), 45 high functioning ASD, and 125 TD individuals were included in this study. After assessing the T1-weighted images, 97 schizophrenia spectrum (26 UHR, 17 FEP, and 64 schizophrenia), 36 ASD, and 106 TD scans were analyzed. The ages of the subjects ranged between 14 and 60 years old (y.o.) for schizophrenia (mean ± SD: 29.8 ± 10.1), 20–44 y.o. for ASD (mean ± SD: 30.1 ± 6.7), 16–60 y.o., for TD (mean ± SD: 29.1 ± 6.0), 16–28 y.o. for UHR (mean ± SD: 20.9 ± 3.1), and 17–34 y.o. for FEP (mean ± SD: 23.5 ± 5.2). Individuals with ASD were all males, those with schizophrenia, TD, UHR, and FEP were of mixed sex. The participants were mostly right-handed (ASD, right (R): 28/left (L): 3/mixed (M): 5; TD R: 104/L: 0/M: 2; schizophrenia R: 55/L: 1/M: 8, UHR R: 23/L: 0/M: 3; FEP R: 16/L: 0/M: 1) (Table 1). All participants are ethnically Japanese and were recruited at The University of Tokyo Hospital. The diagnostic criteria for schizophrenia, and ASD and inclusion and exclusion criteria can be found elsewhere^29–31^, additionally, a comprehensive explanation was added to the supplemental materials. The ethical review board of The University of Tokyo Hospital approved this study (Nos. 397 and 2226). All participants gave written informed consent before their participation.

**Table 1:** Demographic characteristics of the participants.

Variables mean (SD)	ASD [*N* = 36]	Schi [*N* = 64]	TD [*N* = 106]	UHR [*N* = 26]	FEP [*N* = 17]	*P* value [ASD/Schi)	*P* value [ASD/TD]	*P* value [Schi/TD]
Age [years]	30.1 (6.7)	29.8 (10.1)	29.1 (6)	20.9 (3.1)	23.5 (5.2)	0.87	0.42	0.59
Sex [M/F]	36/0	37/27	59/47	15/11	12/5	<0.001	<0.001	0.78
Handedness [R/L/M]	[28/3/5]	[55/1/8]	[104/0/2]	[23/0/3]	[16/0/1]	0.239	<0.001	0.007
*ADI-R*								
Social	14.7 (6.2)							
Communication	11.9 (3.8)							
RRB	4.2 (2)							
*AQ*								
SS	8.1 (2.1)							
AS	7.5 (1.8)							
AD	6.2 (2.3)							
Communication	7.7 (2.2)							
Imagination	7 (2)							
*PANSS*								
PS		14.5 (4.9)		12.9 (3.9)	12.9 (4.8)			
NS		18.8 (6)		16.3 (6.4)	17.9 (4.8)			
GS		34.2 (9.1)		31.8 (8.9)	33.4 (8.5)			
GAF		47.2 (14.8)		54.3 (18.6)	48.4 (14)			

*ASD* autism spectrum disorder, *TD* typically developing, *Schi* schizophrenia, *UHR* ultra-high risk for psychosis, *FEP* first-episode psychosis, *SD* standard deviation, *N* sample size, *M* male, *F* female, *R* right, *L* left, *M* mixed, ADI-R autism diagnostic interview- revised, *RRB* restricted and repetitive behavior, *AQ* autism quotient, *SS* social skills, *AS* attention switching, *AD* attention to details, *PANSS* positive and negative syndrome scale, *PS* positive symptoms, *NS* negative symptoms, *GS* general symptoms, *GAF* global assessment of functioning, *P* value set at *P* = 0.05.

### MRI acquisition

The structural MRI images for all of the subjects were acquired using a 3.0-T MRI scanner (GeneralElectric Healthcare, Signa HDxt. v14.0, Milwaukee, Wisconsin), with a standard 8-channel head coil for signal reception. The T1-weighted structural brain images were collected using a three-dimensional Fourier-transform fast-spoiled gradient recalled acquisition with steady state, because it affords excellent contrast between the gray and white matter (repetition time = 6.80 ms, echo time = 1.94 ms, flip angle = 20°, slice thickness = 1.0 mm, field of view = 240 mm, matrix = 256 × 256, number of axial slices = 176). The participant’s head was fixed with foam pads to minimize movement. A trained neuroradiologist (O.A., W.G., or H.T.) checked the scans and found no gross abnormalities in any of the subjects. Magnetic field inhomogeneity in our scanner was monitored with daily quality control. In order to ensure that the images were of appropriate quality, the scans of all subjects were visually examined, slice by slice across all orthogonal directions before any image processing step. The scans were performed between the year 2010 and 2013.

### Data processing

#### Imaging

The structural MRI scans from all subjects were processed with the same procedure, using the FreeSurfer image analysis suite v.6.0 (http://surfer.nmr.mgh.harvard.edu/). This processing step was performed using recon-all pipeline with the default settings. The details of this procedure^32–34^, can be found in the supplemental materials. Even though the FreeSurfer morphometric procedures have been shown to be accurate and reliable^35^, we implemented additional quality assurance steps. Enhancing NeuroImaging Genetics through Meta-Analysis wrapper script (http://enigma.ini.usc.edu/protocols/imaging-protocols/), was employed after the FreeSurfer processing steps for quality assurance. Subsequently, a visual check was performed on the images to investigate whether there was any sort of abnormality, and manual edits were applied when necessary. When edits were not possible, the scans were discarded from the study. The FreeSurfer output, i.e., cortical thickness (150 regions), surface area (150 regions), and subcortical volume (36 regions) were later used as feature groups in the classification models described in detail in the supplemental materials (Table S1).

#### Quality control and feature engineering

First, the data were checked for missing values, and subjects with any missing value, were excluded from the analysis. Second, the outliers of each group were detected though the interquartile range method and were removed before the start of the analysis. The total number of excluded subjects throughout the study preprocessing steps were 9 ASD, 16 schizophrenia, 8 FEP, and 19 TD subjects. The features included in each group can be found as a table in the Supplemental material (Table S1).

Before the features were used in the classification process, they were standardized using StandardScalar, part of scikit-learn (SKLearn), by removing the mean and scaling to unit variance. StandardScalar was first applied on the training data set and was then reapplied later with the same transformation on the testing set. These sets were randomly selected based on the train/test split function in SKLearn (See “Classification architecture”).

### Classification architecture

All the analyses were implemented using Python v2.7 available at (http://www.python.org) and SkLearn v.0.19.1^36^, a machine learning library for Python. The data was split into training (80%) and testing (20%) sets using the train/test split function in SKLearn. The test set was not used until the very end to assess the performance of the classifiers. StandardScalar was then applied as described in the “Quality control and feature engineering” section. Furthermore, dimensionality reduction was performed using principle component analysis (PCA). PCA utilizes linear dimensionality reduction by using the data’s singular value decomposition to project it to a lower dimensional space^37^. Moreover, as the features are expected to be collinear, PCA also helps to overcome this multicollinearity problem by producing orthogonal features made from the linear combination of the input vectors, i.e., principle components. PCA was performed inside a pipeline which allows setting different parameters and combines several steps that can be cross validated together. This pipeline was implemented inside GridSearchCV, with a tenfold cross-validation, which performs an exhaustive search over the assigned parameters to construct the best possible classifier using a combination of optimal parameter values. Fine-tuning the classifiers entailed using different parameter combinations inside GridSearchCV. The parameters producing a classification with the best performance were chosen, and the model was fit to the entire training set using those parameter values. All the classifiers utilized in this study were fine-tuned and had the same overall architecture. As the sample size of each group is unbalanced, we used the “class_weight” parameter and set it to “balanced”, to ensure that we had more balanced classes. This parameter option works by weighing classes inversely proportional to their frequency. The classifiers used in our study are logistic regression (LR), support vector machine (SVM), random forest (RF), adaptive boosting (AdaB), decision tree (DT), and k-nearest neighbor (kNN). Several classifiers have been selected to avoid bias toward the use of a particular classifier^28^, and to compare their performance on our data. The classifiers were run with several subjects and feature group combinations. Only those classifiers that showed relatively high accuracy, with no signs of overfitting, were reported in the manuscript. Four classification runs were performed with each classifier; one multiclass classification (schizophrenia/ASD/TD), and three binary classifications (schizophrenia/ASD, ASD/TD, and schizophrenia/TD). The code is available upon request.

### Classifiers

#### Logistic regression

The logistic function, a core part of the LR, is a sigmoid function that can take a real number and transforms it into a value between 0 and 1, producing a “S”-shaped curve. LR uses the maximum likelihood estimation method to estimate the model coefficients. It is typically used for binary classification problems, but a multiclass classification is also possible, for example, through the one-vs.-rest scheme.

### Decision tree

The DT method uses a non-parametric supervised learning approach to solve both regression and classification problems. DT uses a tree representation where each test on an attribute is represented by an internal node, and each leaf denotes a class label. Thus, DT can learn certain decision rules inferred from the features used to build a model that predicts the value of the target variable.

### Random forest

RF is an ensemble learning method, consisting of several DTs, that can be used for classification and regression. Ensemble methods are algorithms that incorporate more than one type of algorithm. RF works by constructing a number of DT classifiers which learn and make predictions independently, and outputs a combined single prediction that is the same or better than the output made by the previously constructed DT classifiers.

### SVM classifier

SVM is a supervised discriminative classification method that uses the features belonging to several labeled training examples to construct hyperplanes, high-dimensional planes, for optimally separating the data into different groups. The implementation of the C- support vector classification used in our study is based on the library for SVMs (libsvm).

### Adaptive boosting

AdaBoost is an ensemble boosting algorithm that combines a set of “weak” classifiers into a weighted sum to create a stronger more accurate “boosted” classifier. AdaBoost starts by fitting a classifier on the dataset, and then fits the same version of that classifier on the same dataset where the weights of the misclassified instances are modified so that the next classifier is improved to work on the more challenging instances.

### k-Nearest neighbor

kNN is an instance based learning algorithm, and yet another non-parametric method used for classification and regression. The input consists of a feature space with k closest training examples, where k is assigned by the user, and the output is a class membership. An object is assigned to a class that is most common amongst its nearest neighbors, as the nearest neighbors contribute more to the average than the distant ones.

### Classification performance metrics

The chosen indicator of proper classification was not solely based on accuracy. Thus, we further calculated the confusion matrix, recall score (i.e., sensitivity), precision score, and F1/F2 scores. The metrics are described in detail in the supplemental materials.

### Classifier consistency with clinical severity

After the classification was complete, the correctly/incorrectly classified (CC)/(IC) instances from both patient groups were extracted, binarized and correlated with their clinical scores. Their clinical scores were assigned accordingly: autism diagnostic interview-revised (ADI-R) sub-scale (social, communication, and restricted and repetitive behavior (RRB)), and autism quotient (AQ) subscale (social skills (SS), attention switching (AS), attention to detail (AD), communication, and imagination) for the ASD group, and the Positive and negative symptom scale (PANSS), which includes positive symptoms, negative symptoms, and general psychopathology for the schizophrenia and FEP groups, the total scores of these scales were also included. The CC and IC classes were coded as “1” and “0”. The classifiers were also coded “1” through “6” representing LR, SVM, RF, AdaB, DT, and kNN. Then, for each classifier a Point-Biserial correlation was run using Statistical Package for Social Sciences (SPSS) v.20. For the ASD group, the ADI-R and AQ sub- and total scores were correlated with the binarized CC and IC instances. The same procedure was conducted for schizophrenia replacing the ADI-R and AQ scores with PANSS sub- and total-scores. The statistical significance threshold for this study was set at *P* < 0.05. Bonferroni correction was used to correct for multiple comparisons.

### UHR and FEP

After training the classifiers, we tested their performance on a group of 26 individuals with UHR and 17 with FEP. We chose the best performing multiclass classifier, and binary classifier (schizophrenia/TD) for that purpose out of all the runs. The recall score was considered here as it represents the ability of the classifier to find the positive samples. The resulting classes were then binarized and a Point-Biserial correlation, evaluating the association of the classification with the PANSS and the global assessment of functioning (GAF) data 1 year or more after the time of the first MRI scan was performed.

To assess whether medication affected the classification, we ran two independent sample t-tests (one sided) on the antipsychotic dose taken by the FEP and UHR subjects that were classified into schizophrenia or TD using the multiclass classifier.

## Results

### Classifiers

In the multiclass classification, the best results were produced using the cortical thickness feature group, especially using the LR classifier, with an overall accuracy of 69.0% (Table 2).

**Table 2:** Classification between individuals with schizophrenia, ASD, and TD.

TD, ASD, and schizophrenia (cortical thickness)					
Classifier	Score (%)	TD	Schi	ASD	All
Logistic regression	Mean accuracy				69.0
	Recall score (Sensitivity)	70.0	70.5	60.0	
	Specificity	46.8	77.2	89.6	
	Precision score	73.6	70.5	50.0	
	F1/F2 scores	71.7/70.7	70.5	54.5/57.6	

*ASD* autism spectrum disorder, *TD* typically developing, *Schi* schizophrenia, *All* whole brain or all features combined.

In the binary classification model between schizophrenia and ASD, the majority of the classifiers performed well with several feature groups. Classification using the whole brain feature group was best in both SVM and kNN with an accuracy of 75% for both, as well as LR with slightly lower accuracy at 70%. Using the subcortical volume feature group, all of the classifiers showed relatively good accuracy; LR, 75%, SVM, 80%, RF 75%, AdaBoost 75%, and kNN 85%. The surface area feature group did the worst overall, although only LR had good results with 70% accuracy. In classifying based on cortical thickness, AdaBoost performed the best, at 85% accuracy, followed by LR (80%), and SVM (75%) (Table 3).

**Table 3:** Classification between individuals with schizophrenia, and ASD.

		ASD and schizophrenia											
		(Subcortical)			(Surface area)			(Cortical thickness)			(All features)		
Classifier	Score (%)	Schi	ASD	All	Schi	ASD	All	Schi	ASD	All	Schi	ASD	All
Logistic regression	Mean accuracy			75.0			70.0			80.0			70.0
	Recall score	72.7	77.7		72.7	66.6		90.9	66.6		81.8	55.5	
	Precision score	80.0	70.0		72.7	66.6		76.9	85.7		69.2	71.4	
	F1/F2 scores	76.1/74.0	73.6/76.0		72.7	66.6		83.3/87.7	75.0/69.7		75.0/78.9	62.5/58.1	
Support vector machine	Mean accuracy			80.0						75.0			75.0
	Recall score	81.8	77.7					90.9	55.5		90.9	55.5	
	Precision score	81.8	77.7					71.4	83.3		71.4	83.3	
	F1/F2 scores	81.8	77.7								80.0/86.2	66.6/59.5	
Random Forest	Mean accuracy			75.0									
	Recall score	90.9	55.5										
	Precision score	71.4	83.3										
	F1/F2 scores	80.0/86.2	66.6/59.5										
Adaboost	Mean accuracy			75.0						85.0			
	Recall score	81.8	66.6					100.0	66.6				
	Precision score	75.0	75.0					78.0	100.0				
	F1/F2 scores	78.2/80.3	70.5/68.1					88.0/94.8	80.0/71.4				
k-nearest neighbor	Mean accuracy			85.0									75.0
	Recall score	90.9	77.7								90.9	55.5	
	Precision score	83.3	87.5								71.4	83.3	
	F1/F2 scores	86.9/89.2	82.3/79.5								80.0/86.2	66.6/59.5	

*ASD* autism spectrum disorder, *TD* typically developing, *Schi* schizophrenia, *All* whole brain or all features combined.

In addition, to further investigate the performance of the classifiers on the patient population versus the TD group, we performed the following classifications between; ASD and TD, and schizophrenia and TD. In the ASD and TD group, only SVM was able to perform well with an accuracy of 75.8% using the whole brain feature group. In the subcortical volume, both LR, accuracy 72.4% and SVM, accuracy 89.6% showed good performance. Lastly, in cortical thickness, DT had the best performance with an accuracy of 75.8% (Table 4).

**Table 4:** Classification between individuals with ASD and TD.

		ASD and TD											
		(Subcortical)			(Surface area)			(Cortical thickness)			(All features)		
Classifier	Score (%)	Schi	ASD	All	Schi	ASD	All	Schi	ASD	All	Schi	ASD	All
Logistic regression	Mean accuracy			72.4			70.0			80.0			
	Recall score	68.1	85.7		72.7	66.6		90.9	66.6				
	Precision score	93.7	46.1		72.7	66.6		76.9	85.7				
	F1/F2 scores	78.9/72.1	60.0/73.1		72.7	66.6		83.3/87.7	75.0/69.7				
Support vector machine	Mean accuracy			89.6						75.0			75.8
	Recall score	100.0	57.1					90.9	55.5		77.2	71.4	
	Precision score	88.0	100.0					71.4	83.3		89.4	50.0	
	F1/F2 scores	93.6/97.3	72.7/62.5								82.9/79.4	58.8/65.7	
Decision tree	Mean accuracy									75.8			
	Recall score							77.2	71.4				
	Precision score							89.4	50.0				
	F1/F2 scores							82.9/79.4	58.8/65.7				

*ASD* autism spectrum disorder, *TD* typically developing, Schi schizophrenia, *All* whole brain or all features combined.

While in the schizophrenia and TD group, LR performed better than all the other classifiers using the whole brain feature group with 70.5% accuracy. While using subcortical volume, LR accuracy was 64.7%, SVM 67.6%, RF 76.4%, and AdaBoost 73.5%. Lastly, using cortical thickness, only LR, accuracy 67.6% and DT, accuracy 70.5% performed well (Table 5). All of the classifiers’ results, despite accuracy level and overfitting status, in addition to the full metric data such as recall score, F1/F2 scores, and others can be seen in the supplementary material (Fig. S1, Tables S2–S9).

**Table 5:** Classification between individuals with schizophrenia and TD.

		Schizophrenia and TD											
		(Subcortical)			(Surface area)			(Cortical thickness)			(All features)		
Classifier	Score (%)	Schi	ASD	All	Schi	ASD	All	Schi	ASD	All	Schi	ASD	All
Logistic regression	Mean accuracy			64.7						67.6			70.5
	Recall score	65.2	63.6					69.5	63.6		69.5	72.7	
	Precision score	78.9	46.6					80.0	50.0		84.2	53.3	
	F1/F2 scores	71.4/67.5	53.8/59.3					74.4/71.4	56.0/60.3		76.1/72.0	61.5/67.7	
Support vector machine	Mean accuracy			67.6									
	Recall score	73.9	54.5										
	Precision score	77.2	50.0										
	F1/ F2 scores	75.5/74.5	52.1/53.5										
Random forest	Mean accuracy			76.4									
	Recall score	82.6	63.6										
	Precision score	82.6	63.6										
	F1/F2 scores	82.6	63.6										
AdaBoost	Mean accuracy			73.5									
	Recall score	73.9	72.7										
	Precision score	85.0	57.1										
	F1/F2 scores	79.0/75.8	64.0/68.9										
Decision tree	Mean accuracy									70.5			
	Recall score							73.9	63.6				
	Precision score							80.9	53.8				
	F1/F2 scores							77.2/75.2	58.3/61.4				

*ASD* autism spectrum disorder, *TD* typically developing, *Schi* schizophrenia, *All* whole brain or all features combined.

### Classifiers and clinical severity

The results of the Point-Biserial correlation showed that LR, SVM, and DT were highly consistent with the clinical severity of the patients with ASD. LR in ASD for example, showed high consistency with ADI-R’s RRB (*F*(1,46) = 7.91, *P* corrected = 0.021; CC mean = 5.5, SD = 2.0, IC mean = 3.6, SD = 2.4), and AQ’s AD (*F*(1,46) = 8.45, *P* corrected = 0.03; CC mean = 7.2, SD = 2.3, IC mean = 5.2, SD = 1.6). SVM also showed consistency with ADI-R’s communication (*F*(1,39) = 7.73, *P* corrected = 0.024; CC mean = 12.9, SD = 3.1, IC mean = 10.4, SD = 2.0), and RRB (*F*(1,39) = 11.42, *P* corrected = 0.006; CC mean = 5.6, SD = 2.1, IC mean = 3.4, SD = 1.6), and AQ’s imagination (*F*(1,39) = 10.41, *P* corrected = 0.015; CC mean = 6.6, SD = 8.2, IC mean = 8.2, SD = 1.5). Lastly, DT was consistent with ADI-R’s social domain (*F*(1,14) = 8.23, *P* = 0.012–0.036; CC mean = 10.9, SD = 5.5, IC mean = 18.5, SD = 4.2). In schizophrenia, no correlation survived after correcting for multiple comparisons.

### Classifiers and independent samples

The UHR group that was classified using the multiclass classifier resulted in 15 schizophrenia (57.6%), and 11 TD subjects, but none were classified as ASD. The Point-Biserial correlation showed no relationship with either PANSS or GAF data after correcting for multiple comparison. When run with the schizophrenia/TD subcortical classifier, 96.1% of the sample were classified as schizophrenia.

FEP subjects were also classified using the same procedure as the UHR. 70% of the FEP subjects were classified as schizophrenia by the multiclass classifier, 30% as TD, while none as ASD. No correlation was found with either PANSS or GAF data. When run with the schizophrenia/TD subcortical classifier, 100% of the samples were classified as schizophrenia.

For UHR and FEP, we found no significant difference in antipsychotic dose between the patients classified into schizophrenia and those into TD using the multiclass classifier.

## Discussion

To our knowledge, this is the first study to compare cortical thickness, subcortical volume and surface area using multiple machine learning classifiers between schizophrenia, ASD and TD, and investigate how these trained classifiers extrapolate to UHR and FEP. Our findings indicate that, overall, SVM and LR, were the best performing classifiers, producing high accuracy with least overfitting. Second, cortical thickness and subcortical volume were most useful for the classification, compared to surface area. Third, the LR, SVM, and DT’s output were clinically informative as they were consistent with the patients’ clinical severity. Lastly, we showed that a selection of the trained classifiers was able to predict the diagnostic category of UHR and FEP Individuals.

SVM showed a good overall performance, which is consistent with the published literature^24,25,38–44^. It is by far the most utilized machine learning classifier in the field of neuroimaging^5,45,46^. SVM has been used in several studies involving both ASD^41–44^ and schizophrenia^24,25,38–40,47,48^. Part of its strength comes from the ability to make inferences at the level of an individual, which is important in a sample of patients with neuropsychiatric disorders having within group heterogeneity^5^. Moreover, the multivariate nature of SVM allows it to reveal subtle differences in the brain that would otherwise not be detectable through univariate group comparisons^5,49^, which helps its performance.

LR was the only classifier that showed no overfitting in the multiclass classification model. In binary classification, it showed a similar performance to SVM, with good overall accuracy. LR has been used in many neuroimaging studies as well^9,42,50,51^. However, we were unable to find structural MRI studies using LR to classify individuals with ASD and TD. Most of the studies that are published use resting-state functional MRI, and show a similar overall classification accuracy as our study^42,50^. One study classifying individuals with ASD and TD, reported similar results to ours, in which LR and SVM were the best performing classifiers amongst those used (LR, RF, kNN, SVM, linear discriminate analysis, and Naïve Bayes)^9^. In schizophrenia, on the other hand, Greenstein et al.^3^ showed that LR was able to classify schizophrenia subjects with a 73.6% accuracy using 74 anatomical brain sub-regions.

Our results also show that RF, DT, kNN, and AdaB did have high performance, at least in specific runs. These classifiers were shown to be useful in several neuroimaging studies of ASD and schizophrenia^3,4,7,9,52^.

As mentioned previously, cortical thickness and subcortical volume performed better than surface area. Structural volume and cortical thickness features have both shown high accuracy classification in the literature^53^. A previous study compared their classification performance between individuals with ASD and TD, and found that thickness-based diagnostic models outperformed those that are based on volume in most classifiers^28^. In our case, the performance of these feature groups was comparable. Another study, by Katuwal et al., showed that surface area performed worse than subcortical volume, which is consistent with our results, though they also showed that surface area performed better than cortical thickness^54^, which is different from what we report in this study. Cortical thickness’s high overall performance signifies the presence of distinct cortical morphological features that are unique to each diagnostic group. The brain surface area’s stability in adults, where the majority of changes such as neural stem cell proliferation and migration happen during early embryonic development^55^, compared to that of cortical thickness, where early developmental changes continue into adulthood^56^, might have contributed to more distinguishable features in cortical thickness than surface area, which was revealed consistently in the performance of all the classifiers used in our study. A previous study comparing different psychiatric disorders including ASD and schizophrenia found that there is more divergence between disorders in cortical thickness than surface area^57^, which would be another reason why cortical thickness performed better than surface area.

In the same study, by Park et al.^57^, they demonstrate that ASD shows a trend toward an increase in cortical thickness while schizophrenia towards cortical thinning, this might explain why our results exhibited higher performance distinguishing between ASD/TD, than schizophrenia/TD. Using the multiclass classifier, cortical thickness was the only feature group that was able to distinguish between all three patient groups. This is noteworthy as it suggests that cortical thickness might hold information valuable for distinguishing between schizophrenia and ASD, and that the overlap in their symptoms might be less explained by cortical morphological features. Lastly, the results from the whole brain feature group shows that integrating different modalities doesn’t always improve the overall accuracy^58,59^, as the combined features, whole brain, did not perform better than the separate features, e.g., cortical thickness.

Most of the classifiers’ output showed an association with the clinical indices of ASD, especially LR and SVM. LR and SVM were highly associated with both ADI-R and AQ, while DT showed an association with ADI-R only. As for schizophrenia, only DT showed an association with the PANSS’s negative symptoms, general symptoms and total score, however this association did not survive Bonferroni correction. To our knowledge, there are no published studies that have assessed this in schizophrenia or ASD.

The early phase of the disease, such as in the UHR as well as FEP, is an important period that can have an outstanding influence on disorder progression^60^. Early intervention in both has been previously associated with better outcomes^60,61^. The multiclass classifier was run on 26 individuals with UHR and 17 with FEP. The classifier separated the UHR group into schizophrenia and TD, but not ASD. While when the schizophrenia/TD classifier was used, almost all of the subjects (96%) were classified into the schizophrenic group. The results for FEP were very similar to those in the UHR group. It is interesting that even at these early stages in disease progression, UHR and FEP have such structural brain similarities with schizophrenia. Collectively, this mean that UHR and more strongly FEP, have shared structural neurobiological patterns with schizophrenia, but not with autism. This method was used in a previous study, but showed modest generalization when a classifier, that was trained on schizophrenia and TD, was used to classify individuals with FEP^62^. It is plausible that the schizophrenia/TD classifier categorized FEP individuals in the schizophrenia group more than the UHR, since the FEP individuals already had their first psychotic episode. These results shed light on the importance of brain structure in understanding disease progression, especially whether or not the patients had their first psychotic episode.

In our sample, the antipsychotic dose that might have been associated with cortical thickness alterations was not predicted by the multiclass classifier. We could not conduct the same analysis using the schizophrenia/TD classifier, as there are not enough samples classified as TD. Given the variability in dose as well as the modest sample size, we are unable to provide a measurably reliable answer on whether, in general, this would influence the classification.

In summary, we found that SVM and LR were the best performing classifiers. Cortical thickness and subcortical volume based classification had better performance across different diagnostic labels and classifiers than surface area. LR, SVM, and DT were consistent with clinical severity of the patients. UHR and FEP show similar neurobiological pattern as schizophrenia. Our findings provide new knowledge about the best performing classifiers between individuals with schizophrenia, ASD, and TD, and reveal the most informative brain feature groups that contribute to the classification. The results also reveal the clinical relevance of these classifiers, in addition to their importance in predicting the diagnostic category. Lastly, they shed light on the structural brain similarities between FEP, UHR and schizophrenia. The knowledge gained from these feature groups can be extrapolated to their use as biomarkers for future targeted therapeutic interventions as well as predicting patients’ disease trajectory.

Parts of the discussion compared our findings to those that are already published, however it should be taken into consideration that comparisons such as accuracies, and class predictions across studies is not ideal as it may be affected by the number of instances, classifiers used, quality and type of images, feature engineering, and other factors^45^.

## Limitations

This study has some limitations. First, the ASD subjects were all males. This, however, did not seem to affect the classification, as we did not see that individuals with ASD were particularly classified better than the other groups. Second, the present study has a modest sample size, nonetheless comparable to other published studies. Third, as few of the ASD subjects, several of the UHR and FEP, and all of the schizophrenia subjects were medicated, this might have affected the results.

## Supplementary information

Supplementary information
