# Supplementary material for: Machine-learning classification using neuroimaging data in schizophrenia, autism, ultra-high risk and first-episode psychosis
Source: Transl Psychiatry. 2020 Aug 17;10:278. doi: 10.1038/s41398-020-00965-5 (PMC7429957; doi:10.1038/s41398-020-00965-5)
Supplement: Supplementary file 1 — Supplementary information [file 41398_2020_965_MOESM1_ESM.doc]

**Machine learning classification using neuroimaging data in schizophrenia, autism, ultra-high risk and first episode psychosis**

**Supplementary Methods**

**Participants**

The diagnosis for individuals with autism spectrum disorder (ASD) was made using the Diagnostic and Statistical Manual of Mental Disorders-text revision, 4^th^ edition^1^, by an expert child and adolescent psychiatrist, and confirmed using the Japanese version of Autism Diagnostic Interview-Revised (ADI-R)^2^, or Autism Diagnosis Observation Schedule^3^, if the participants did not reach the threshold in the social domain of ADI-R. The eligibility criteria included an intelligence quotient of more than 80. While for schizophrenia the diagnosis was determined according to the Diagnostic and Statistical Manual of Mental Disorders 4^th^ edition (DSM-IV)^4^, by an experienced psychiatrist and confirmed with more than 6-months of follow-up using the Structured Clinical Interview for DSM-IV Axis I Disorder Clinical Version^5^. The Positive and Negative Syndrome Scale was used to evaluate the psychotic symptoms^6^. The diagnosis of the ultra-high risk for psychosis subjects was made according to the Structured Interview for Prodromal Symptoms (SIPS)^7, 8^, while for the first episode psychosis subjects was made using the DSM-IV, with an inclusion criteria comprising a first experience of acute psychosis defined according to the SIPS, antipsychotic medication for less than 16 cumulative weeks, and continuous psychotic symptoms for less than 60 months. Exclusion criteria for all groups consisted of a current or past neurological illness, brain injury and its related cognitive consequences, substance use or addiction, and, additionally, for those with schizophrenia, a history of electroconvulsive therapy, while for typically developing (TD) individuals, a history of psychiatric disorders or family history of an axis I disorder among first degree relatives. All the ASD subjects that entered into the analysis were psychotropic-free, except for three participants, one was medicated with an antipsychotic, one with a selective serotonin reuptake inhibitor and one with a serotonin-norepinephrine reuptake inhibitor. Antipsychotics were used by all the participants with schizophrenia.

**Data processing**

**Imaging**

The FreeSurfer processing pipeline includes motion correction, removal of non-brain tissue^9^, automated Talairach transformation, segmentation of the subcortical white matter and deep gray matter volumetric structures (including the hippocampus, amygdala, caudate, putamen, ventricles)^10, 11^, intensity normalization, tessellation of the gray matter/white matter boundary, automated topology correction, and surface deformation following intensity gradients to optimally place the gray/white and gray/cerebrospinal fluid borders at the location where the greatest shift in intensity defines the transition to the other tissue class. Since a 3T MR scanner was used to scan the participants, a “-3T” flag was included to utilize the 3T-spesific *NU* intensity correction parameters.

**Feature groups**

In order to understand which of the feature groups, i.e. cortical thickness, surface area, and subcortical volume, in addition to all the features combined (termed “whole brain” henceforth), best describes the decision of the classifiers, the following 4 feature groups (i~iv) were used. The classification was performed using i) age, sex (one hot encoded), age x sex, and cortical thickness only (150 features), ii) age, sex (one hot encoded), age x sex, and surface area only (150 features), iii) age, sex (one hot encoded), age x sex, and subcortical volume only (38 features), and iv) age, sex (one hot encoded), age x sex, and whole brain i.e. cortical thickness, surface area and subcortical volume (334 features). A list of all the features included can be found in table S1.

Another run was performed with the same features as above excluding age, sex and their interaction.

**Classification performance metrics**

Before defining each of these metrics, it is important to mention some of their components. A true positive (TP) is the number of the correctly identified subjects as belonging to their respective class label. A true negative (TN) is the number of correctly identified subjects that they belong to another class label. False positive (FP, or type I error) is the number of subjects that were classified as belonging to a particular class label when in fact they do not. False negative (FN, or type II error) is the number of subjects that were classified as not belonging to a particular class label when in fact, they do.

*Accuracy*

Measures how “successful” the machine learning model was in its prediction. This measure is often misleading to follow on its own.

***Accuracy =***
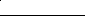


*Confusion matrix*

The confusion matrix helps to visualize the classification outcome. The columns represent the actual instances of the classes while the rows represent the predicted. Several information could be extracted from the confusion matrix, directly such as type I and type II errors, and indirectly through simple calculations, for example the sensitivity (recall score), specificity, precision, F1 and F2 scores.

*Sensitivity (Recall score)*

The recall score, also referred to as “sensitivity”, is the ratio of the true positives over the sum of the true positives and false negatives. It can provide a measure of the proportion of the actual positives that were identified by the model.

***Recall score =***
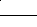


*Specificity*

Specificity is the ratio of true negatives over the sun of true negatives and false positives. It is defined as the proportion of actual negatives that were correctly predicted as negatives. The higher values of this metric mean higher numbers of true negatives.

***Specificity =***
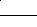


*Precision score*

The precision score is the ratio of the true positives over the sum of the true positives and false positives. It gives information about the correct positive predictions out of all the positive predictions.

***Precision scare =***
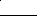


*F1/F2 scores*

F1 score is the test’s harmonic mean of both the recall and precision. The recall score ranges between 0 (worst score), and 1 (best possible score) where we have prefect recall and precision. It provides a measure of accuracy that is different from the traditional measure of accuracy mentioned above. F2 score is a weighted average of the precision and recall. It weights recall higher than precision. It can be used when it is more important to classify correctly as many positive instances as possible rather than having the highest number of correct classification.

***F1 =***
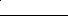
***, F2 =***
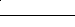


*F1/F2 score combination (A Clinically relevant metric)*

As mentioned in the previous section, there are scenarios where it is more important to have as many correctly classified positive instances than to maximize the number of correct classification. This is also clinically helpful, as we would rather identify all the patients -with few healthy individuals that were falsely identified as patients- rather than miss any of the subjects in the patient group. In other words, we accept to have a classifier that has some false positive (type I error) in the TD group while maximizing the true positive of the patient group. Thus, we propose the following: in the multiclass and binary classification models, of the patient groups versus TD individuals, or one patient group versus the other, F2 score was adopted as a measure of the classifier performance for the patient group and F1 score was adopted for the TD group. We used the F1 score for the TD group as we don't have a strong preference to detect these individuals as much as the patients. The final measure included an average of both the F1 score of the TD group and the F2 score of the patient groups when the classification was between patients and TD, and an average of the F2 scores of the patient groups when the classification was between the patient groups i.e. ASD and schizophrenia. In the case of a multiclass classification problem, the average of the F1 score from the TD group and the F2 scores from the first patient group as well as the second patient group was calculated.

**Over- and under- sampling**

Over- and under-sampling were performed using the RandomOverSampler and RandomUnderSampler, part of the imblearn over-/under- sampling package respectively. RandomOverSampler over samples the minority class by choosing samples from it at randome with replacement. While RandomUnderSampler under samples the majority class by picking samples from it at random with or without replacement. We ran the analysis twice, one with RandomUnderSample’s default setting i.e. without replacement, and one with replacement.

**Supplementary Results**

**Over- and under- sampling**

Performing over- and under- sampling as described in the supplementary methods section did not enhance the classification.

**Clinically relevant metrics**

The clinically relevant metrics described previously were calculated for each of the classifiers, and only the classifiers with the highest scores were presented. In the multi-class classification, which includes the ASD, schizophrenia and TD, LR had an F1 score of 71.79% for TD, F2 score of 70.58% for schizophrenia and 57.69% for ASD with a total average of 66.68%. In the ASD and schizophrenia group, kNN classifier based on subcortical volume features showed the highest average score 84.41%, F2 score of schizophrenia 89.28% and that of ASD is 79.54%. In the ASD and TD group classification, SVM using the subcortical volume features performed best with an average of 78.05%; F2 score of ASD 62.5% and F1 score of TD 93.61%. Lastly in the schizophrenia and TD group, AdaBoost performed best using subcortical volume features with an average score of 74.01%; F2 score of 68.96% for schizophrenia and F1 score of 79.06% for TD.

**Performing the analysis without age and sex**

The results of the classification accuracy without including age and sex as features showed modest changes, improvement and deterioration of performance, from the results we report in the main manuscript. These can be seen in tables S6~S9.

**References**

1. American Psychiatric Association. Diagnostic and Statistical Manual of Mental Disorders-text revised. 4th ed. (DSM-IV-TR). American Psychiatric publishing Inc. 2000

2. Lord C, Rutter M, Le Couteur A. Autism Diagnostic Interview-Revised: a revised version of a diagnostic interview for caregivers of individuals with possible pervasive developmental disorders. Journal of autism and developmental disorders Oct 1994;24(5):659-685.

3. Lord C. et al. Autism diagnostic observation schedule: a standardized observation of communicative and social behavior. Journal of autism and developmental disorders Jun 1989;19(2):185-212.

4. American Psychiatric Association. Diagnostic and Statistical Manual of Mental Disorders. 4th ed. (DSM-IV). American Psychiatric Press, Washington DC. 1994

5. First MB, Spitzer RL, Gibbon M, Williams JBW. Structured Clinical Interview for DSM-IV Axis Disorder: Clinical Version (SCID-CV). American Psychiatric Press, Washington DC. 1997

6. Kay SR, Fiszbein A, Opler LA. The positive and negative syndrome scale (PANSS) for schizophrenia. Schizophrenia bulletin 1987;13(2):261-276.

7. Miller TJ. et al. Symptom assessment in schizophrenic prodromal states. The Psychiatric quarterly. 1999;70:273-287.

8. McGlashan TH, Miller TJ, Woods SW. Structural Interview for Prodromal Syndromes (v3.0). PRIME Research Clinic, Yale School of Medicine, New Haven. 2001

9. Segonne F. et al. A hybrid approach to the skull stripping problem in MRI. NeuroImage Jul 2004;22(3):1060-1075.

10. Fischl B. et al. Whole brain segmentation: automated labeling of neuroanatomical structures in the human brain. Neuron Jan 31

11. Fischl B. et al. Sequence-independent segmentation of magnetic resonance images. NeuroImage 2004;23 Suppl 1:S69-84. 1:S69-84.

**Figure S1. The overall accuracy of classification of ASD, schizophrenia and typically developing individuals using all the classifiers.**

**
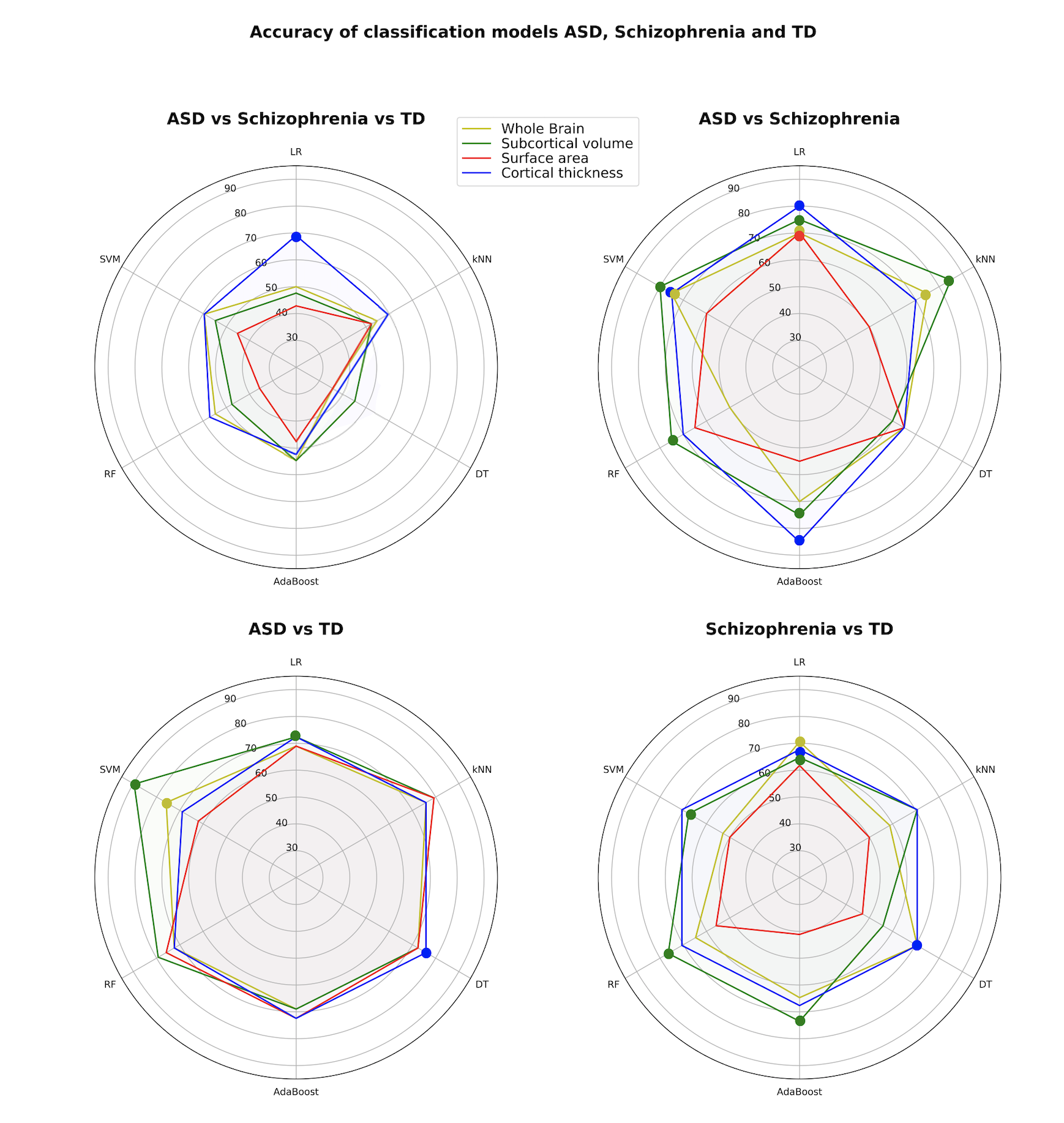
**

LR= Logistic regression, DT= decision tree, RF= random forest, SVM= support vector machine, AdaB= adaptive boosting, and kNN= k nearest neighbor, numbers represent percentages, the absence of a dot on the spider figure indicates an over fitting of the classifier, colors indicate feature groups.

Table S1. The features used in the subcortical volume, surface area, and cortical thickness feature groups.

| **Cortical thickness features** | **Surface area features** | **Subcortical volume** |
| --- | --- | --- |
| lh_G&S_frontomargin_thickness | lh_G&S_frontomargin_area | Left-Lateral-Ventricle |
| lh_G&S_occipital_inf_thickness | lh_G&S_occipital_inf_area | Left-Inf-Lat- Ventricle |
| lh_G&S_paracentral_thickness | lh_G&S_paracentral_area | Left-Cerebellum-White-Matter |
| lh_G&S_subcentral_thickness | lh_G&S_subcentral_area | Left-Cerebellum-Cortex |
| lh_G&S_transv_frontopol_thickness | lh_G&S_transv_frontopol_area | Left-Thalamus-Proper |
| lh_G&S_cingul-Ant_thickness | lh_G&S_cingul-Ant_area | Left-Caudate |
| lh_G&S_cingul-Mid-Ant_thickness | lh_G&S_cingul-Mid-Ant_area | Left-Putamen |
| lh_G&S_cingul-Mid-Post_thickness | lh_G&S_cingul-Mid-Post_area | Left-Pallidum |
| lh_G_cingul-Post-dorsal_thickness | lh_G_cingul-Post-dorsal_area | 3rd-Ventricle |
| lh_G_cingul-Post-ventral_thickness | lh_G_cingul-Post-ventral_area | 4th-Ventricle |
| lh_G_cuneus_thickness | lh_G_cuneus_area | Brain-Stem |
| lh_G_front_inf-Opercular_thickness | lh_G_front_inf-Opercular_area | Left-Hippocampus |
| lh_G_front_inf-Orbital_thickness | lh_G_front_inf-Orbital_area | Left-Amygdala |
| lh_G_front_inf-Triangul_thickness | lh_G_front_inf-Triangul_area | CSF |
| lh_G_front_middle_thickness | lh_G_front_middle_area | Left-Accumbens-area |
| lh_G_front_sup_thickness | lh_G_front_sup_area | Left-VentralDC |
| lh_G_Ins_lg&S_cent_ins_thickness | lh_G_Ins_lg&S_cent_ins_area | Left-choroid-plexus |
| lh_G_insular_short_thickness | lh_G_insular_short_area | Right-Lateral-Ventricle |
| lh_G_occipital_middle_thickness | lh_G_occipital_middle_area | Right-Inf-Lat- Ventricle |
| lh_G_occipital_sup_thickness | lh_G_occipital_sup_area | Right-Cerebellum-White-Matter |
| lh_G_oc-temp_lat-fusifor_thickness | lh_G_oc-temp_lat-fusifor_area | Right-Cerebellum-Cortex |
| lh_G_oc-temp_med-Lingual_thickness | lh_G_oc-temp_med-Lingual_area | Right-Thalamus-Proper |
| lh_G_oc-temp_med-Parahip_thickness | lh_G_oc-temp_med-Parahip_area | Right-Caudate |
| lh_G_orbital_thickness | lh_G_orbital_area | Right-Putamen |
| lh_G_pariet_inf-Angular_thickness | lh_G_pariet_inf-Angular_area | Right-Pallidum |
| lh_G_pariet_inf-Supramar_thickness | lh_G_pariet_inf-Supramar_area | Right-Hippocampus |
| lh_G_parietal_sup_thickness | lh_G_parietal_sup_area | Right-Amygdala |
| lh_G_postcentral_thickness | lh_G_postcentral_area | Right-Accumbens-area |
| lh_G_precentral_thickness | lh_G_precentral_area | Right-VentralDC |
| lh_G_precuneus_thickness | lh_G_precuneus_area | Right-choroid-plexus |
| lh_G_rectus_thickness | lh_G_rectus_area | Optic-Chiasm |
| lh_G_subcallosal_thickness | lh_G_subcallosal_area | CC_Posterior |
| lh_G_temp_sup-G_T_transv_thickness | lh_G_temp_sup-G_T_transv_area | CC_Mid_Posterior |
| lh_G_temp_sup-Lateral_thickness | lh_G_temp_sup-Lateral_area | CC_Central |
| lh_G_temp_sup-Plan_polar_thickness | lh_G_temp_sup-Plan_polar_area | CC_Mid_Anterior |
| lh_G_temp_sup-Plan_tempo_thickness | lh_G_temp_sup-Plan_tempo_area | CC_Anterior |
| lh_G_temporal_inf_thickness | lh_G_temporal_inf_area |  |
| lh_G_temporal_middle_thickness | lh_G_temporal_middle_area |
| lh_Lat_Fis-ant-Horizont_thickness | lh_Lat_Fis-ant-Horizont_area |
| lh_Lat_Fis-ant-Vertical_thickness | lh_Lat_Fis-ant-Vertical_area |
| lh_Lat_Fis-post_thickness | lh_Lat_Fis-post_area |
| lh_Pole_occipital_thickness | lh_Pole_occipital_area |
| lh_Pole_temporal_thickness | lh_Pole_temporal_area |
| lh_S_calcarine_thickness | lh_S_calcarine_area |
| lh_S_central_thickness | lh_S_central_area |
| lh_S_cingul-Marginalis_thickness | lh_S_cingul-Marginalis_area |
| lh_S_circular_insula_ant_thickness | lh_S_circular_insula_ant_area |
| lh_S_circular_insula_inf_thickness | lh_S_circular_insula_inf_area |
| lh_S_circular_insula_sup_thickness | lh_S_circular_insula_sup_area |
| lh_S_collat_transv_ant_thickness | lh_S_collat_transv_ant_area |
| lh_S_collat_transv_post_thickness | lh_S_collat_transv_post_area |
| lh_S_front_inf_thickness | lh_S_front_inf_area |
| lh_S_front_middle_thickness | lh_S_front_middle_area |
| lh_S_front_sup_thickness | lh_S_front_sup_area |
| lh_S_interm_prim-Jensen_thickness | lh_S_interm_prim-Jensen_area |
| lh_S_intrapariet&P_transv_thickness | lh_S_intrapariet&P_transv_area |
| lh_S_oc_middle&Lunatus_thickness | lh_S_oc_middle&Lunatus_area |
| lh_S_oc_sup&transversal_thickness | lh_S_oc_sup&transversal_area |
| lh_S_occipital_ant_thickness | lh_S_occipital_ant_area |
| lh_S_oc-temp_lat_thickness | lh_S_oc-temp_lat_area |
| lh_S_oc-temp_med&Lingual_thickness | lh_S_oc-temp_med&Lingual_area |
| lh_S_orbital_lateral_thickness | lh_S_orbital_lateral_area |
| lh_S_orbital_med-olfact_thickness | lh_S_orbital_med-olfact_area |
| lh_S_orbital-H_Shaped_thickness | lh_S_orbital-H_Shaped_area |
| lh_S_parieto_occipital_thickness | lh_S_parieto_occipital_area |
| lh_S_pericallosal_thickness | lh_S_pericallosal_area |
| lh_S_postcentral_thickness | lh_S_postcentral_area |
| lh_S_precentral-inf-part_thickness | lh_S_precentral-inf-part_area |
| lh_S_precentral-sup-part_thickness | lh_S_precentral-sup-part_area |
| lh_S_suborbital_thickness | lh_S_suborbital_area |
| lh_S_subparietal_thickness | lh_S_subparietal_area |
| lh_S_temporal_inf_thickness | lh_S_temporal_inf_area |
| lh_S_temporal_sup_thickness | lh_S_temporal_sup_area |
| lh_S_temporal_transverse_thickness | lh_S_temporal_transverse_area |
| rh_G&S_frontomargin_thickness | rh_G&S_frontomargin_area |
| rh_G&S_occipital_inf_thickness | rh_G&S_occipital_inf_area |
| rh_G&S_paracentral_thickness | rh_G&S_paracentral_area |
| rh_G&S_subcentral_thickness | rh_G&S_subcentral_area |
| rh_G&S_transv_frontopol_thickness | rh_G&S_transv_frontopol_area |
| rh_G&S_cingul-Ant_thickness | rh_G&S_cingul-Ant_area |
| rh_G&S_cingul-Mid-Ant_thickness | rh_G&S_cingul-Mid-Ant_area |
| rh_G&S_cingul-Mid-Post_thickness | rh_G&S_cingul-Mid-Post_area |
| rh_G_cingul-Post-dorsal_thickness | rh_G_cingul-Post-dorsal_area |
| rh_G_cingul-Post-ventral_thickness | rh_G_cingul-Post-ventral_area |
| rh_G_cuneus_thickness | rh_G_cuneus_area |
| rh_G_front_inf-Opercular_thickness | rh_G_front_inf-Opercular_area |
| rh_G_front_inf-Orbital_thickness | rh_G_front_inf-Orbital_area |
| rh_G_front_inf-Triangul_thickness | rh_G_front_inf-Triangul_area |
| rh_G_front_middle_thickness | rh_G_front_middle_area |
| rh_G_front_sup_thickness | rh_G_front_sup_area |
| rh_G_Ins_lg&S_cent_ins_thickness | rh_G_Ins_lg&S_cent_ins_area |
| rh_G_insular_short_thickness | rh_G_insular_short_area |
| rh_G_occipital_middle_thickness | rh_G_occipital_middle_area |
| rh_G_occipital_sup_thickness | rh_G_occipital_sup_area |
| rh_G_oc-temp_lat-fusifor_thickness | rh_G_oc-temp_lat-fusifor_area |
| rh_G_oc-temp_med-Lingual_thickness | rh_G_oc-temp_med-Lingual_area |
| rh_G_oc-temp_med-Parahip_thickness | rh_G_oc-temp_med-Parahip_area |
| rh_G_orbital_thickness | rh_G_orbital_area |
| rh_G_pariet_inf-Angular_thickness | rh_G_pariet_inf-Angular_area |
| rh_G_pariet_inf-Supramar_thickness | rh_G_pariet_inf-Supramar_area |
| rh_G_parietal_sup_thickness | rh_G_parietal_sup_area |
| rh_G_postcentral_thickness | rh_G_postcentral_area |
| rh_G_precentral_thickness | rh_G_precentral_area |
| rh_G_precuneus_thickness | rh_G_precuneus_area |
| rh_G_rectus_thickness | rh_G_rectus_area |
| rh_G_subcallosal_thickness | rh_G_subcallosal_area |
| rh_G_temp_sup-G_T_transv_thickness | rh_G_temp_sup-G_T_transv_area |
| rh_G_temp_sup-Lateral_thickness | rh_G_temp_sup-Lateral_area |
| rh_G_temp_sup-Plan_polar_thickness | rh_G_temp_sup-Plan_polar_area |
| rh_G_temp_sup-Plan_tempo_thickness | rh_G_temp_sup-Plan_tempo_area |
| rh_G_temporal_inf_thickness | rh_G_temporal_inf_area |
| rh_G_temporal_middle_thickness | rh_G_temporal_middle_area |
| rh_Lat_Fis-ant-Horizont_thickness | rh_Lat_Fis-ant-Horizont_area |
| rh_Lat_Fis-ant-Vertical_thickness | rh_Lat_Fis-ant-Vertical_area |
| rh_Lat_Fis-post_thickness | rh_Lat_Fis-post_area |
| rh_Pole_occipital_thickness | rh_Pole_occipital_area |
| rh_Pole_temporal_thickness | rh_Pole_temporal_area |
| rh_S_calcarine_thickness | rh_S_calcarine_area |
| rh_S_central_thickness | rh_S_central_area |
| rh_S_cingul-Marginalis_thickness | rh_S_cingul-Marginalis_area |
| rh_S_circular_insula_ant_thickness | rh_S_circular_insula_ant_area |
| rh_S_circular_insula_inf_thickness | rh_S_circular_insula_inf_area |
| rh_S_circular_insula_sup_thickness | rh_S_circular_insula_sup_area |
| rh_S_collat_transv_ant_thickness | rh_S_collat_transv_ant_area |
| rh_S_collat_transv_post_thickness | rh_S_collat_transv_post_area |
| rh_S_front_inf_thickness | rh_S_front_inf_area |
| rh_S_front_middle_thickness | rh_S_front_middle_area |
| rh_S_front_sup_thickness | rh_S_front_sup_area |
| rh_S_interm_prim-Jensen_thickness | rh_S_interm_prim-Jensen_area |
| rh_S_intrapariet&P_transv_thickness | rh_S_intrapariet&P_transv_area |
| rh_S_oc_middle&Lunatus_thickness | rh_S_oc_middle&Lunatus_area |
| rh_S_oc_sup&transversal_thickness | rh_S_oc_sup&transversal_area |
| rh_S_occipital_ant_thickness | rh_S_occipital_ant_area |
| rh_S_oc-temp_lat_thickness | rh_S_oc-temp_lat_area |
| rh_S_oc-temp_med&Lingual_thickness | rh_S_oc-temp_med&Lingual_area |
| rh_S_orbital_lateral_thickness | rh_S_orbital_lateral_area |
| rh_S_orbital_med-olfact_thickness | rh_S_orbital_med-olfact_area |
| rh_S_orbital-H_Shaped_thickness | rh_S_orbital-H_Shaped_area |
| rh_S_parieto_occipital_thickness | rh_S_parieto_occipital_area |
| rh_S_pericallosal_thickness | rh_S_pericallosal_area |
| rh_S_postcentral_thickness | rh_S_postcentral_area |
| rh_S_precentral-inf-part_thickness | rh_S_precentral-inf-part_area |
| rh_S_precentral-sup-part_thickness | rh_S_precentral-sup-part_area |
| rh_S_suborbital_thickness | rh_S_suborbital_area |
| rh_S_subparietal_thickness | rh_S_subparietal_area |
| rh_S_temporal_inf_thickness | rh_S_temporal_inf_area |
| rh_S_temporal_sup_thickness | rh_S_temporal_sup_area |
| rh_S_temporal_transverse_thickness | rh_S_temporal_transverse_area |

rh = right hemisphere; lh = left hemisphere; S = Sulcus; G = gyrus; sup = superior; inf = inferior; ant = anterior; lat = lateral; transv = transverse; cent = central; mid = middle; lg = long; temp = temporal; oc = occipital; ins = insula; prim = primus; fis = fissure; CSF = cerebrospinal fluid

Table S2. Logistic regression, support vector machine, random forest, adaboost, decision tree and k-nearest neighbor classification between individuals with schizophrenia, ASD, and TD using subcortical volume, surface area, cortical thickness and whole brain feature groups (Sex and age included).

|  | | Schizophrenia, ASD, TD | | | | | | | | | | | | | | | |
| --- | --- | --- | --- | --- | --- | --- | --- | --- | --- | --- | --- | --- | --- | --- | --- | --- | --- |
|  |  |  | (Subcortical) | | |  | (Surface area) | | |  | (Cortical thickness) | | |  | (All features) | | |
| Classifier | Score (%) | Schi | ASD | TD | All | Schi | ASD | TD | All | Schi | ASD | TD | All | Schi | ASD | TD | All |
| Logistic regression | Mean accuracy |  |  |  | 47.6 |  |  |  | 42.8 |  |  |  | 69.0 |  |  |  | 50.0 |
|  | Recall score | 41.1 | 40.0 | 55.0 |  | 35.2 | 20.0 | 55.0 |  | 70.5 | 60.0 | 70.0 |  | 35.2 | 20.0 | 70.0 |  |
|  | Precision score | 58.3 | 20.0 | 55.0 |  | 37.5 | 16.6 | 55.0 |  | 70.5 | 50.0 | 73.6 |  | 50.0 | 16.6 | 58.3 |  |
|  | F1/ F2 scores | 48.2/ 43.7 | 26.6/ 33.3 | 55.0/ 55.0 |  | 36.3/　35.7 | 18.1/　19.2 | 55.0/ 55.0 |  | 70.5/ 70.5 | 54.5/ 57.6 | 71.7/ 70.7 |  | 41.3/ 37.5 | 18.1/ 19.2 | 63.6/ 67.3 |  |
| Support vector machine | Mean accuracy |  |  |  | 54.7 |  |  |  | 45.2 |  |  |  | 59.5 |  |  |  | 59.5 |
|  | Recall score | 52.9 | 20.0 | 65.0 |  | 35.2 | 0.0 | 65.0 |  | 47.0 | 20.0 | 80.0 |  | 41.1 | 40.0 | 80.0 |  |
|  | Precision score | 60.0 | 12.5 | 68.4 |  | 42.8 | 0.0 | 56.5 |  | 72.7 | 50.0 | 55.1 |  | 77.8 | 40.0 | 57.1 |  |
|  | F1/ F2 scores | 56.2/ 54.2 | 15.3/ 17.8 | 66.7/ 65.6 |  | 38.7/ 36.5 | 0.0/ 0.0 | 60.4/ 63.1 |  | 57.1/ 50.6 | 28.5/ 22.7 | 65.3/ 73.3 |  | 53.8/ 45.4 | 40.0 | 66.7/ 74.0 |  |
| Random Forest | Mean accuracy |  |  |  | 47.6 |  |  |  | 35.7 |  |  |  | 57.1 |  |  |  | 54.7 |
|  | Recall score | 58.8 | 00 | 95.0 |  | 29.4 | 0.0 | 50.0 |  | 23.5 | 0.0 | 100.0 |  | 29.4 | 0.0 | 90.0 |  |
|  | Precision score | 50.0 | 0.0 | 47.5 |  | 38.4 | 0.0 | 38.4 |  | 57.1 | 0.0 | 60.6 |  | 71.4 | 0.0 | 52.9 |  |
|  | F1/ F2 scores | 10.5/ 7.1 | 0.0/ 0.0 | 63.3/ 79.1 |  | 33.3/ 30.8 | 0.0/ 0.0 | 43.4/ 47.1 |  | 33.3/ 26.6 | 0.0/ 0.0 | 75.4/ 88.4 |  | 41.6/ 33.3 | 0.0/ 0.0 | 66.7/ 78.9 |  |
| Adaboost | Mean accuracy |  |  |  | 54.7 |  |  |  | 47.6 |  |  |  | 52.3 |  |  |  | 54.7 |
|  | Recall score | 35.2 | 20.0 | 80.0 |  | 0.0 | 0.0 | 100.0 |  | 41.1 | 0.0 | 75.0 |  | 23.5 | 0.0 | 95.0 |  |
|  | Precision score | 85.7 | 25.0 | 51.6 |  | 0.0 | 0.0 | 50.0 |  | 77.8 | 0.0 | 53.5 |  | 66.7 | 0.0 | 55.8 |  |
|  | F1/ F2 scores | 50.0/ 40.0 | 22.2/ 20.8 | 62.7/ 72.0 |  | 0.0/ 0.0 | 0.0/ 0.0 | 66.7/ 83.3 |  | 53.8/45.4 | 0.0 | 62.5/69.4 |  | 34.7/ 27.0 | 0.0/ 0.0 | 70.3/ 83.3 |  |
| Decision Tree | Mean accuracy |  |  |  | 45.2 |  |  |  | 35.7 |  |  |  | 38.0 |  |  |  | 35.7 |
|  | Recall score | 47.0 | 20.0 | 50.0 |  | 35.2 | 0.0 | 45.0 |  | 29.4 | 20.0 | 50.0 |  | 23.5 | 0.0 | 55.0 |  |
|  | Precision score | 50.0 | 16.6 | 50.0 |  | 42.8 | 0.0 | 39.1 |  | 41.6 | 12.5 | 45.4 |  | 26.6 | 0.0 | 47.8 |  |
|  | F1/ F2 scores | 48.4/ 47.6 | 18.1/ 19.2 | 50.0/ 50.0 |  | 38.7/ 36.5 | 0.0/ 0.0 | 41.8/ 43.6 |  | 34.4/ 31.2 | 15.3/ 17.8 | 47.6/ 49.0 |  | 25.0/ 24.0 | 0.0/ 0.0 | 51.1/ 53.3 |  |
| k- nearest neighbor | Mean accuracy |  |  |  | 52.3 |  |  |  | 52.38 |  |  |  | 59.5 |  |  |  | 54.7 |
|  | Recall score | 17.6 | 0.0 | 95.0 |  | 35.2 | 20.0 | 75.0 |  | 35.2 | 40.0 | 85.0 |  | 17.6 | 20.0 | 95.0 |  |
|  | Precision score | 75.0 | 0.0 | 51.3 |  | 46.1 | 33.3 | 57.6 |  | 60.0 | 100.0 | 56.7 |  | 75.0 | 100.0 | 51.3 |  |
|  | F1/ F2 scores | 28.5/ 20.8 | 0.0/ 0.0 | 66.7/ 81.1 |  | 40.0/ 37.0 | 25.0/ 21.7 | 65.2/ 70.7 |  | 44.4/　38.4 | 57.1/ 45.4 | 68.0/ 77.2 |  | 28.5/ 20.8 | 33.3/ 23.8 | 66.7/ 81.1 |  |

ASD = Autism spectrum disorder; TD = Typically developing; Schi = Schizophrenia; All = Whole brain or all features combined.

Table S3. Logistic regression, support vector machine, random forest, adaboost, decision tree and k-nearest neighbor classification between individuals with ASD and TD using subcortical volume, surface area, cortical thickness and whole brain feature groups (Sex and age included).

|  | | ASD, TD | | | | | | | | | | | |
| --- | --- | --- | --- | --- | --- | --- | --- | --- | --- | --- | --- | --- | --- |
|  |  | (Subcortical) | | | (Surface area) | | | (Cortical thickness) | | | (All features) | | |
| Classifier | Score (%) | ASD | TD | All | ASD | TD | All | ASD | TD | All | ASD | TD | All |
| Logistic regression | Mean accuracy |  |  | 72.4 |  |  | 68.9 |  |  | 72.4 |  |  | 68.9 |
|  | Recall score | 85.7 | 68.1 |  | 14.2 | 86.3 |  | 28.5 | 86.36 |  | 28.5 | 81.8 |  |
|  | Precision score | 46.1 | 93.7 |  | 25.0 | 76.0 |  | 40.0 | 79.16 |  | 33.3 | 78.2 |  |
|  | F1/ F2 scores | 60.0/73.1 | 78.9/72.1 |  | 18.1/15.6 | 80.8/84.0 |  | 33.33/30.30 | 82.6/84.8 |  | 30.7/29.4 | 80.0/81.0 |  |
| Support vector machine | Mean accuracy |  |  | 89.6 |  |  | 62.0 |  |  | 68.9 |  |  | 75.8 |
|  | Recall score | 57.1 | 100.0 |  | 0.0 | 81.8 |  | 14.2 | 86.3 |  | 71.4 | 77.2 |  |
|  | Precision score | 100.0 | 88.0 |  | 0.0 | 72.0 |  | 25.0 | 76.0 |  | 50.0 | 89.4 |  |
|  | F1/ F2 scores | 72.7/62.5 | 93.6/97.3 |  | 0.0/ 0.0 | 76.5/79.6 |  | 18.1/15.6 | 80.8/84.0 |  | 58.8/65.7 | 82.9/79.4 |  |
| Random Forest | Mean accuracy |  |  | 79.3 |  |  | 75.8 |  |  | 72.4 |  |  | 72.4 |
|  | Recall score | 28.5 | 95.4 |  | 0.0 | 100.0 |  | 28.5 | 86.3 |  | 14.2 | 90.9 |  |
|  | Precision score | 66.7 | 80.7 |  | 0.0 | 75.8 |  | 40.0 | 79.1 |  | 33.3 | 76.9 |  |
|  | F1/ F2 scores | 40.0/32.2 | 87.7/92.1 |  | 0.0/ 0.0 | 86.2/94.0 |  | 33.3/30.3 | 82.6/84.8 |  | 20.0/26.1 | 83.3/87.7 |  |
| Adaboost | Mean accuracy |  |  | 68.9 |  |  | 72.4 |  |  | 72.4 |  |  | 68.9 |
|  | Recall score | 14.2 | 86.3 |  | 14.2 | 90.9 |  | 28.5 | 86.3 |  | 0.0 | 90.9 |  |
|  | Precision score | 25.0 | 76.0 |  | 33.3 | 76.9 |  | 40.0 | 79.1 |  | 0.0 | 74.0 |  |
|  | F1/ F2 scores | 18.1/15.6 | 80.8/84.0 |  | 20.0/16.1 | 83.3/87.7 |  | 33.3/30.3 | 82.6/84.8 |  | 0.0/ 0.0 | 81.6/86.9 |  |
| Decision Tree | Mean accuracy |  |  | 72.4 |  |  | 72.4 |  |  | 75.8 |  |  | 72.4 |
|  | Recall score | 28.5 | 86.3 |  | 28.5 | 86.3 |  | 71.4 | 77.2 |  | 28.5 | 86.3 |  |
|  | Precision score | 40.0 | 79.1 |  | 40 | 79.1 |  | 50 | 89.4 |  | 40.0 | 79.1 |  |
|  | F1/ F2 scores | 33.3/30.3 | 82.6/84.8 |  | 33.3/30.3 | 82.6/84.8 |  | 58.8/65.7 | 82.9/79.4 |  | 33.3/30.3 | 82.6/ 84.8 |  |
| k- nearest neighbor | Mean accuracy |  |  | 79.3 |  |  | 79.3 |  |  | 75.8 |  |  | 75.8 |
|  | Recall score | 28.5 | 95.4 |  | 14.2 | 100.0 |  | 42.8 | 86.3 |  | 14.2 | 95.4 |  |
|  | Precision score | 66.7 | 80.7 |  | 100.0 | 78.5 |  | 50.0 | 82.6 |  | 50.0 | 77.7 |  |
|  | F1/ F2 scores | 40.0/32.2 | 87.5/92.0 |  | 25.0/17.2 | 88.0/94.8 |  | 46.1/44.1 | 84.4/85.5 |  | 22.2/16.6 | 85.7/91.3 |  |

ASD = Autism spectrum disorder; TD = Typically developing; Schi = Schizophrenia; All = Whole brain or all features combined.

Table S4. Logistic regression, support vector machine, random forest, adaboost, decision tree and k-nearest neighbor classification between individuals with schizophrenia and TD using subcortical volume, surface area, cortical thickness and whole brain feature groups (Sex and age included).

|  | | Schizophrenia, TD | | | | | | | | | | | |
| --- | --- | --- | --- | --- | --- | --- | --- | --- | --- | --- | --- | --- | --- |
|  |  | (Subcortical) | | | (Surface area) | | | (Cortical thickness) | | | (All features) | | |
| Classifier | Score (%) | Schi | TD | All | Schi | TD | All | Schi | TD | All | Schi | TD | All |
| Logistic regression | Mean accuracy |  |  | 64.7 |  |  | 61.7 |  |  | 67.6 |  |  | 70.5 |
|  | Recall score | 63.64 | 65.2 |  | 45.4 | 69.5 |  | 63.6 | 69.5 |  | 72.7 | 69.5 |  |
|  | Precision score | 46.6 | 78.9 |  | 41.6 | 72.7 |  | 50.0 | 80.0 |  | 53.3 | 84.2 |  |
|  | F1/ F2 scores | 53.8/59.3 | 71.4/67.5 |  | 43.4/44.6 | 71.1/70.1 |  | 56.0/60.3 | 74.4/71.4 |  | 61.5/ 67.7 | 76.1/72.0 |  |
| Support vector machine | Mean accuracy |  |  | 67.6 |  |  | 50.0 |  |  | 70.5 |  |  | 52.9 |
|  | Recall score | 54.5 | 73.9 |  | 36.3 | 56.5 |  | 54.5 | 78.2 |  | 72.7 | 43.4 |  |
|  | Precision score | 50.0 | 77.2 |  | 28.5 | 65.0 |  | 54.5 | 78.2 |  | 38.0 | 76.9 |  |
|  | F1/ F2 scores | 52.1/53.5 | 75.5/74.5 |  | 32.0/34.4 | 60.4/58.0 |  | 54.5/ 54.5 | 78.2/ 78.2 |  | 50.0/ 61.5 | 55.5/47.6 |  |
| Random Forest | Mean accuracy |  |  | 76.4 |  |  | 55.8 |  |  | 70.5 |  |  | 64.7 |
|  | Recall score | 63.6 | 82.6 |  | 9.0 | 78.2 |  | 27.2 | 91.3 |  | 9.0 | 91.3 |  |
|  | Precision score | 63.6 | 82.6 |  | 16.6 | 64.2 |  | 60.0 | 72.4 |  | 33.3 | 67.7 |  |
|  | F1/ F2 scores | 63.6/ 63.6 | 82.6/ 82.6 |  | 11.7/100.0 | 70.5/75.0 |  | 37.5/30.6 | 80.7/86.7 |  | 14.2/10.6 | 77.7/85.3 |  |
| Adaboost | Mean accuracy |  |  | 73.5 |  |  | 41.1 |  |  | 67.6 |  |  | 64.7 |
|  | Recall score | 72.7 | 73.9 |  | 27.2 | 47.8 |  | 45.4 | 78.2 |  | 36.3 | 78.2 |  |
|  | Precision score | 57.1 | 85.0 |  | 20.0 | 57.8 |  | 50.0 | 75.0 |  | 44.4 | 72.0 |  |
|  | F1/ F2 scores | 64.0/68.9 | 79.0/75.8 |  | 23.0/25.4 | 52.3/49.5 |  | 47.6/46.2 | 76.5/77.5 |  | 40.0/ 37.7 | 75.0/76.9 |  |
| Decision Tree | Mean accuracy |  |  | 55.8 |  |  | 47.0 |  |  | 70.5 |  |  | 70.5 |
|  | Recall score | 81.8 | 43.4 |  | 36.3 | 52.1 |  | 63.6 | 73.9 |  | 45.4 | 82.6 |  |
|  | Precision score | 40.9 | 83.3 |  | 26.6 | 63.1 |  | 53.8 | 80.9 |  | 55.6 | 76.0 |  |
|  | F1/ F2 scores | 54.5/68.1 | 57.1/48.0 |  | 30.7/33.8 | 57.1/54.0 |  | 58.3/61.4 | 77.2/75.2 |  | 50.0/ 47.1 | 79.1/81.1 |  |
| k- nearest neighbor | Mean accuracy |  |  | 70.58 |  |  | 50.0 |  |  | 70.5 |  |  | 58.8 |
|  | Recall score | 18.1 | 95.6 |  | 36.3 | 56.5 |  | 36.3 | 86.9 |  | 27.2 | 73.9 |  |
|  | Precision score | 66.7 | 70.9 |  | 28.5 | 65.0 |  | 57.1 | 74.0 |  | 33.3 | 68.0 |  |
|  | F1/ F2 scores | 28.5/21.2 | 81.4/89.4 |  | 32.0/34.4 | 60.4/58.0 |  | 44.4/39.2 | 80.0/84.0 |  | 30.0/28.3 | 70.8/72.6 |  |

ASD = Autism spectrum disorder; TD = Typically developing; Schi = Schizophrenia; All = Whole brain or all features combined.

Table S5. Logistic regression, support vector machine, random forest, adaboost, decision tree and k-nearest neighbor classification between individuals with schizophrenia, and ASD using subcortical volume, surface area, cortical thickness and whole brain feature groups (Sex and age included).

|  | | Schizophrenia, ASD | | | | | | | | | | | |
| --- | --- | --- | --- | --- | --- | --- | --- | --- | --- | --- | --- | --- | --- |
|  |  | (Subcortical) | | | (Surface area) | | | (Cortical thickness) | | | (All features) | | |
| Classifier | Score (%) | Schi | ASD | All | Schi | ASD | All | Schi | ASD | All | Schi | ASD | All |
| Logistic regression | Mean accuracy |  |  | 75.0 |  |  | 70.0 |  |  | 80.0 |  |  | 70.0 |
|  | Recall score | 72.7 | 77.7 |  | 72.7 | 66.7 |  | 90.9 | 66.7 |  | 81.81 | 55.5 |  |
|  | Precision score | 80.0 | 70.0 |  | 72.7 | 66.7 |  | 76.9 | 85.7 |  | 69.2 | 71.4 |  |
|  | F1/ F2 scores | 76.1/74.0 | 73.6/76.0 |  | 72.7/ 72.7 | 66.7/ 66.7 |  | 83.3/87.7 | 75.0/69.7 |  | 75.0/78.9 | 62.5/58.1 |  |
| Support vector machine | Mean accuracy |  |  | 80.0 |  |  | 60.0 |  |  | 75.0 |  |  | 75.0 |
|  | Recall score | 81.8 | 77.7 |  | 90.9 | 22.2 |  | 90.9 | 55.5 |  | 90.9 | 55.5 |  |
|  | Precision score | 81.8 | 77.7 |  | 58.8 | 66.7 |  | 71.4 | 83.3 |  | 71.4 | 83.3 |  |
|  | F1/ F2 scores | 81.8/ 81.8 | 77.7/ 77.7 |  | 71.4/81.9 | 33.3/25.6 |  | 80.0/86.2 | 66.7/59.5 |  | 80.0/86.2 | 66.7/59.5 |  |
| Random Forest | Mean accuracy |  |  | 75.0 |  |  | 65.0 |  |  | 70.0 |  |  | 50.0 |
|  | Recall score | 90.9 | 55.5 |  | 100.0 | 22.2 |  | 100.0 | 33.3 |  | 81.8 | 11.1 |  |
|  | Precision score | 71.4 | 83.3 |  | 61.1 | 100.0 |  | 64.7 | 100.0 |  | 52.9 | 33.3 |  |
|  | F1/ F2 scores | 80.0/86.2 | 66.7/59.5 |  | 75.8/88.7 | 36.3/26.3 |  | 78.5/90.1 | 50.0/38.4 |  | 64.2/73.7 | 16.6/12.8 |  |
| Adaboost | Mean accuracy |  |  | 75.0 |  |  | 55.0 |  |  | 85.0 |  |  | 70.0 |
|  | Recall score | 81.8 | 66.7 |  | 90.9 | 11.1 |  | 100.0 | 66.7 |  | 90.9 | 44.4 |  |
|  | Precision score | 75.0 | 75.0 |  | 55.5 | 50.0 |  | 78.0 | 100.0 |  | 66.7 | 80.0 |  |
|  | F1/ F2 scores | 78.2/80.3 | 70.5/68.1 |  | 68.9/80.6 | 18.1/13.1 |  | 88.0/94.8 | 80.0/71.4 |  | 76.9/84.7 | 57.1/48.7 |  |
| Decision Tree | Mean accuracy |  |  | 60.0 |  |  | 65.0 |  |  | 65.0 |  |  | 65.0 |
|  | Recall score | 63.6 | 55.5 |  | 90.9 | 33.3 |  | 81.8 | 44.4 |  | 81.8 | 44.4 |  |
|  | Precision score | 63.6 | 55.5 |  | 62.5 | 75.0 |  | 64.2 | 66.7 |  | 64.2 | 66.7 |  |
|  | F1/ F2 scores | 63.6/ 63.6 | 55.5/ 55.5 |  | 74.0/83.3 | 46.1/37.5 |  | 72.0/77.5 | 53.3/47.6 |  | 72.0/77.5 | 53.3/47.6 |  |
| k- nearest neighbor | Mean accuracy |  |  | 85.0 |  |  | 50.0 |  |  | 70.0 |  |  | 75.0 |
|  | Recall score | 90.9 | 77.7 |  | 72.7 | 22.2 |  | 100.0 | 33.3 |  | 90.9 | 55.5 |  |
|  | Precision score | 83.3 | 87.5 |  | 53.3 | 40.0 |  | 64.7 | 100.0 |  | 71.4 | 83.3 |  |
|  | F1/ F2 scores | 86.9/89.2 | 82.3/79.5 |  | 61.5/67.7 | 28.5/24.3 |  | 78.5/90.1 | 50.0/38.4 |  | 80.0/86.2 | 66.7/59.5 |  |

ASD = Autism spectrum disorder; TD = Typically developing; Schi = Schizophrenia; All = Whole brain or all features combined.

Table S6. Logistic regression, support vector machine, random forest, adaboost, decision tree and k-nearest neighbor classification between individuals with schizophrenia, ASD, and TD using subcortical volume, surface area, cortical thickness and whole brain feature groups (Sex and age excluded).

|  | | Schizophrenia, ASD, TD | | | | | | | | | | | | | | | |
| --- | --- | --- | --- | --- | --- | --- | --- | --- | --- | --- | --- | --- | --- | --- | --- | --- | --- |
|  |  |  | (Subcortical) | | |  | (Surface area) | | |  | (Cortical thickness) | | |  | (All features) | | |
| Classifier | Score (%) | Schi | ASD | TD | All | Schi | ASD | TD | All | Schi | ASD | TD | All | Schi | ASD | TD | All |
| Logistic regression | Mean accuracy |  |  |  | 47.6 |  |  |  | 42.8 |  |  |  | 50.0 |  |  |  | 61.9 |
|  | Recall score | 35.2 | 40.0 | 60.0 |  | 29.4 | 40.0 | 55.0 |  | 47.0 | 0.0 | 65.0 |  | 47.0 | 20.0 | 85.0 |  |
|  | Precision score | 60.0 | 18.1 | 57.1 |  | 35.7 | 33.3 | 50.0 |  | 53.3 | 0.0 | 48.1 |  | 72.7 | 25.5 | 62.9 |  |
|  | F1/ F2 scores | 44.4/ 38.4 | 25.0/ 32.2 | 58.5/ 59.4 |  | 32.2/　30.4 | 36.3/　38.4 | 52.3/ 53.9 |  | 50.0/ 48.1 | 0.0/ 0.0 | 55.3/ 60.7 |  | 57.1/ 50.6 | 22.2/ 20.8 | 72.3/ 79.4 |  |
| Support vector machine | Mean accuracy |  |  |  | 54.7 |  |  |  | 47.6 |  |  |  | 57.1 |  |  |  | 54.7 |
|  | Recall score | 17.6 | 20.0 | 95.0 |  | 5.8 | 0.0 | 95.0 |  | 41.1 | 20.0 | 80.0 |  | 41.1 | 40.0 | 70 |  |
|  | Precision score | 100.0 | 16.7 | 57.5 |  | 50.0 | 0.0 | 48.7 |  | 63.3 | 100.0 | 53.3 |  | 58.3 | 40.0 | 56.0 |  |
|  | F1/ F2 scores | 30.0/ 21.1 | 18.1/ 19.2 | 71.6/ 84.0 |  | 10.5/ 7.1 | 0.0/ 0.0 | 64.4/ 79.8 |  | 50.0/ 44.3 | 33.3/ 23.8 | 64.0/ 72.3 |  | 48.2/ 43.7 | 40.0/ 40.0 | 62.2/ 66.7 |  |
| Random Forest | Mean accuracy |  |  |  | 52.3 |  |  |  | 52.3 |  |  |  | 47.6 |  |  |  | 54.7 |
|  | Recall score | 17.6 | 0.0 | 95.0 |  | 17.6 | 0.0 | 95.0 |  | 23.5 | 0.0 | 80.0 |  | 23.5 | 0.0 | 95.0 |  |
|  | Precision score | 60.0 | 0.0 | 51.3 |  | 50 | 0.0 | 54.2 |  | 57.1 | 0.0 | 47.0 |  | 66.7 | 0.0 | 55.8 |  |
|  | F1/ F2 scores | 27.2/ 20.5 | 0.0/ 0.0 | 66.7/ 81.1 |  | 26.0/ 20.2 | 0.0/ 0.0 | 69.0/ 82.6 |  | 33.3/ 26.6 | 0.0/ 0.0 | 59.2/ 70.1 |  | 34.7/ 27.0 | 0.0/ 0.0 | 70.3/ 83.3 |  |
| Adaboost | Mean accuracy |  |  |  | 52.3 |  |  |  | 47.6 |  |  |  | 35.7 |  |  |  | 50.0 |
|  | Recall score | 17.6 | 20.0 | 90.0 |  | 23.5 | 0.0 | 80.0 |  | 17.6 | 20.0 | 55.0 |  | 5.8 | 0.0 | 100.0 |  |
|  | Precision score | 75.0 | 20.0 | 54.5 |  | 57.1 | 0.0 | 53.3 |  | 27.2 | 33.3 | 39.2 |  | 100.0 | 0.0 | 48.7 |  |
|  | F1/ F2 scores | 28.5/ 20.8 | 20.0/ 20.0 | 67.9/ 79.6 |  | 33.3/ 26.7 | 0.0/ 0.0 | 64.0/ 72.7 |  | 21.4/ 18.9 | 25.0/ 21.7 | 45.8/ 50.9 |  | 11.1/ 7.2 | 0.0/ 0.0 | 65.5/ 82.6 |  |
| Decision Tree | Mean accuracy |  |  |  | 47.6 |  |  |  | 47.6 |  |  |  | 38.0 |  |  |  | 42.8 |
|  | Recall score | 35.2 | 40.0 | 60.0 |  | 35.2 | 0.0 | 70.0 |  | 11.7 | 60.0 | 55.0 |  | 23.5 | 20.0 | 65.0 |  |
|  | Precision score | 50.0 | 20.0 | 60.0 |  | 46.1 | 0.0 | 58.3 |  | 25.0 | 27.2 | 47.8 |  | 36.3 | 20.0 | 50.0 |  |
|  | F1/ F2 scores | 41.3/ 37.5 | 26.7/ 33.3 | 60.0/ 60.0 |  | 40.0/ 37.0 | 0.0/ 0.0 | 63.6/ 67.3 |  | 16.0/ 13.1 | 37.5/ 48.3 | 51.1/ 53.3 |  | 28.5/ 25.3 | 20.0/ 20.0 | 56.5/ 61.3 |  |
| k- nearest neighbor | Mean accuracy |  |  |  | 52.3 |  |  |  | 50.0 |  |  |  | 52.3 |  |  |  | 54.7 |
|  | Recall score | 23.5 | 0.0 | 90.0 |  | 35.2 | 0.0 | 75.0 |  | 35.2 | 20.0 | 75.0 |  | 29.4 | 0.0 | 90.0 |  |
|  | Precision score | 100.0 | 0.0 | 52.9 |  | 60.0 | 0.0 | 51.7 |  | 46.1 | 100.0 | 53.5 |  | 71.4 | 0.0 | 51.4 |  |
|  | F1/ F2 scores | 38.0/ 27.8 | 0.0/ 0.0 | 66.7/ 78.9 |  | 44.4/ 38.4 | 0.0/ 0.0 | 61.22/ 68.8 |  | 40.0/　37.0 | 33.3/ 23.8 | 62.5/ 69.4 |  | 41.6/ 33.3 | 0.0/ 0.0 | 65.4/ 78.2 |  |

ASD = Autism spectrum disorder; TD = Typically developing; Schi = Schizophrenia; All = Whole brain or all features combined.

Table S7. Logistic regression, support vector machine, random forest, adaboost, decision tree and k-nearest neighbor classification between individuals with ASD and TD using subcortical volume, surface area, cortical thickness and whole brain feature groups (Sex and age excluded).

|  | | ASD, TD | | | | | | | | | | | |
| --- | --- | --- | --- | --- | --- | --- | --- | --- | --- | --- | --- | --- | --- |
|  |  | (Subcortical) | | | (Surface area) | | | (Cortical thickness) | | | (All features) | | |
| Classifier | Score (%) | ASD | TD | All | ASD | TD | All | ASD | TD | All | ASD | TD | All |
| Logistic regression | Mean accuracy |  |  | 65.5 |  |  | 72.4 |  |  | 72.4 |  |  | 79.3 |
|  | Recall score | 42.8 | 72.7 |  | 28.5 | 86.3 |  | 28.5 | 86.36 |  | 71.4 | 81.8 |  |
|  | Precision score | 33.3 | 80.0 |  | 40.0 | 79.1 |  | 40.0 | 79.16 |  | 55.6 | 90 |  |
|  | F1/ F2 scores | 37.5/40.5 | 76.1/74.0 |  | 33.3/30.3 | 82.6/84.8 |  | 33.33/30.30 | 82.6/84.8 |  | 62.5/67.5 | 85.7/83.3 |  |
| Support vector machine | Mean accuracy |  |  | 79.3 |  |  | 62.0 |  |  | 68.9 |  |  | 75.8 |
|  | Recall score | 28.5 | 95.4 |  | 0 | 81.8 |  | 14.2 | 86.3 |  | 28.5 | 90.9 |  |
|  | Precision score | 66.7 | 80.7 |  | 0 | 72.0 |  | 25.0 | 76.0 |  | 50.0 | 80.0 |  |
|  | F1/ F2 scores | 40/32.2 | 87.5/92.1 |  | 0 | 76.5/79.6 |  | 18.1/15.6 | 80.8/84.0 |  | 36.3/31.5 | 85.1/88.4 |  |
| Random Forest | Mean accuracy |  |  | 72.4 |  |  | 75.8 |  |  | 75.8 |  |  | 72.4 |
|  | Recall score | 14.2 | 90.9 |  | 0 | 100.0 |  | 0.0 | 100.0 |  | 14.2 | 90.9 |  |
|  | Precision score | 33.3 | 76.9 |  | 0 | 75.8 |  | 0.0 | 75.8 |  | 33.3 | 76.9 |  |
|  | F1/ F2 scores | 20.0/16.1 | 83.3/87.7 |  | 0 | 86.2/94.0 |  | 0.0/0.0 | 86.2/94.0 |  | 20.0/16.1 | 83.3/87.7 |  |
| Adaboost | Mean accuracy |  |  | 72.4 |  |  | 75.8 |  |  | 75.8 |  |  | 68.9 |
|  | Recall score | 0.0 | 95.4 |  | 14.2 | 95.4 |  | 28.5 | 90.9 |  | 57.1 | 72.7 |  |
|  | Precision score | 0.0 | 75.0 |  | 50.0 | 77.8 |  | 50.0 | 80.0 |  | 40.0 | 84.2 |  |
|  | F1/ F2 scores | 0.0/0.0 | 84.0/90.5 |  | 22.2/16.7 | 85.7/91.3 |  | 36.3/31.2 | 85.1/88.4 |  | 47.0/52.6 | 78.0/74.7 |  |
| Decision Tree | Mean accuracy |  |  | 65.5 |  |  | 62.0 |  |  | 55.1 |  |  | 75.8 |
|  | Recall score | 42.8 | 72.7 |  | 14.2 | 77.2 |  | 28.5 | 63.6 |  | 57.1 | 81.8 |  |
|  | Precision score | 33.3 | 80.0 |  | 16.7 | 73.9 |  | 20.0 | 73.68 |  | 50.0 | 85.7 |  |
|  | F1/ F2 scores | 37.5/40.5 | 76.1/74.0 |  | 15.3/14.7 | 75.7/76.5 |  | 23.5/26.3 | 68.2/65.4 |  | 53.3/55.6 | 83.7/82.5 |  |
| k- nearest neighbor | Mean accuracy |  |  | 82.7 |  |  | 75.8 |  |  | 72.4 |  |  | 79.3 |
|  | Recall score | 42.8 | 95.4 |  | 0.0 | 100.0 |  | 14.2 | 90.9 |  | 28.5 | 95.4 |  |
|  | Precision score | 75.0 | 84.0 |  | 0.0 | 75.8 |  | 33.3 | 76.9 |  | 66.7 | 80.7 |  |
|  | F1/ F2 scores | 54.4/46.8 | 89.3/92.9 |  | 0.0/0.0 | 86.2/94.0 |  | 20/16.1 | 83.3/87.7 |  | 40.0/32.2 | 85.7/92.1 |  |

ASD = Autism spectrum disorder; TD = Typically developing; Schi = Schizophrenia; All = Whole brain or all features combined.

Table S8. Logistic regression, support vector machine, random forest, adaboost, decision tree and k-nearest neighbor classification between individuals with schizophrenia and TD using subcortical volume, surface area, cortical thickness and whole brain feature groups (Sex and age excluded).

|  | | Schizophrenia, TD | | | | | | | | | | | |
| --- | --- | --- | --- | --- | --- | --- | --- | --- | --- | --- | --- | --- | --- |
|  |  | (Subcortical) | | | (Surface area) | | | (Cortical thickness) | | | (All features) | | |
| Classifier | Score (%) | Schi | TD | All | Schi | TD | All | Schi | TD | All | Schi | TD | All |
| Logistic regression | Mean accuracy |  |  | 50.0 |  |  | 44.1 |  |  | 70.5 |  |  | 70.5 |
|  | Recall score | 45.4 | 52.1 |  | 36.3 | 47.8 |  | 72.7 | 69.5 |  | 63.6 | 73.9 |  |
|  | Precision score | 31.2 | 66.7 |  | 25.0 | 61.1 |  | 53.3 | 84.2 |  | 53.8 | 80.9 |  |
|  | F1/ F2 scores | 37.0/ 41.6 | 58.5/ 54.5 |  | 29.6/33.3 | 53.6/50.0 |  | 61.5/ 67.7 | 76.1/ 72.0 |  | 58.3/ 61.4 | 77.2/ 75.2 |  |
| Support vector machine | Mean accuracy |  |  | 70.5 |  |  | 47.0 |  |  | 67.6 |  |  | 67.6 |
|  | Recall score | 27.2 | 91.3 |  | 18.1 | 60.8 |  | 54.5 | 73.9 |  | 63.6 | 69.5 |  |
|  | Precision score | 60.0 | 72.4 |  | 18.1 | 60.8 |  | 50.0 | 77.2 |  | 50.0 | 80.0 |  |
|  | F1/ F2 scores | 37.5/ 30.6 | 80.7/ 86.7 |  | 18.1/18.1 | 60.8/60.8 |  | 52.1/ 53.5 | 75.7/ 74.5 |  | 56.0/ 60.3 | 74.4/ 71.4 |  |
| Random Forest | Mean accuracy |  |  | 61.7 |  |  | 50.0 |  |  | 58.8 |  |  | 70.5 |
|  | Recall score | 45.4 | 69.5 |  | 27.2 | 60.8 |  | 54.5 | 60.8 |  | 45.4 | 82.6 |  |
|  | Precision score | 41.6 | 72.7 |  | 25.0 | 63.6 |  | 40.0 | 73.6 |  | 55.6 | 76.0 |  |
|  | F1/ F2 scores | 43.4/ 44.6 | 71.1/ 70.1 |  | 26.0/26.7 | 62.2/61.4 |  | 46.1/ 50.8 | 66.7/ 63.0 |  | 50.0/ 47.1 | 79.1/ 81.1 |  |
| Adaboost | Mean accuracy |  |  | 58.8 |  |  | 67.6 |  |  | 70.5 |  |  | 79.4 |
|  | Recall score | 36.3 | 69.5 |  | 36.3 | 82.6 |  | 45.4 | 82.6 |  | 63.6 | 86.9 |  |
|  | Precision score | 36.3 | 69.5 |  | 50.0 | 73.0 |  | 55.5 | 76.0 |  | 70.0 | 83.3 |  |
|  | F1/ F2 scores | 36.3/ 36.3 | 69.5/ 69.5 |  | 42.1/38.4 | 77.5/80.5 |  | 50.0/ 47.1 | 79.1/ 81.1 |  | 66.7/ 64.8 | 85.1/ 86.2 |  |
| Decision Tree | Mean accuracy |  |  | 52.9 |  |  | 44.1 |  |  | 64.7 |  |  | 70.5 |
|  | Recall score | 36.3 | 60.8 |  | 36.3 | 47.8 |  | 72.7 | 60.8 |  | 54.5 | 78.2 |  |
|  | Precision score | 30.7 | 66.7 |  | 25.0 | 61.1 |  | 47.0 | 82.3 |  | 54.5 | 78.2 |  |
|  | F1/ F2 scores | 33.3/ 35.0 | 63.6/ 61.9 |  | 29.6/33.3 | 53.6/50.0 |  | 57.1/65.5 | 70.0/64.2 |  | 54.5/ 54.5 | 78.2/ 78.2 |  |
| k- nearest neighbor | Mean accuracy |  |  | 82.3 |  |  | 47.0 |  |  | 73.5 |  |  | 52.9 |
|  | Recall score | 45.4 | 100.0 |  | 45.4 | 47.8 |  | 45.4 | 86.9 |  | 18.1 | 69.5 |  |
|  | Precision score | 100.0 | 79.3 |  | 29.4 | 64.7 |  | 62.5 | 76.9 |  | 22.2 | 64.0 |  |
|  | F1/ F2 scores | 62.5/ 51.0 | 88.4/ 95.0 |  | 35.7/ 40.9 | 55.0/ 50.4 |  | 52.6/ 48.0 | 81.6/ 84.7 |  | 20.0/ 18.8 | 66.7/ 68.3 |  |

ASD = Autism spectrum disorder; TD = Typically developing; Schi = Schizophrenia; All = Whole brain or all features combined.

Table S9. Logistic regression, support vector machine, random forest, adaboost, decision tree and k-nearest neighbor classification between individuals with schizophrenia and ASD, using subcortical volume, surface area, cortical thickness and whole brain feature groups (Sex and age excluded).

|  | | Schizophrenia, ASD | | | | | | | | | | | |
| --- | --- | --- | --- | --- | --- | --- | --- | --- | --- | --- | --- | --- | --- |
|  |  | (Subcortical) | | | (Surface area) | | | (Cortical thickness) | | | (All features) | | |
| Classifier | Score (%) | Schi | ASD | All | Schi | ASD | All | Schi | ASD | All | Schi | ASD | All |
| Logistic regression | Mean accuracy |  |  | 75.0 |  |  | 65.0 |  |  | 80.0 |  |  | 75.0 |
|  | Recall score | 72.7 | 77.7 |  | 72.7 | 55.6 |  | 90.9 | 66.7 |  | 90.9 | 55.6 |  |
|  | Precision score | 80.0 | 70.0 |  | 66.7 | 62.5 |  | 76.9 | 85.7 |  | 71.4 | 83.3 |  |
|  | F1/ F2 scores | 76.1/ 74.0 | 73.6/ 76.0 |  | 69.5/ 71.4 | 58.8/ 56.8 |  | 83.3/87.7 | 75.0/69.7 |  | 80.0/ 86.2 | 66.7/ 59.5 |  |
| Support vector machine | Mean accuracy |  |  | 85.0 |  |  | 75.0 |  |  | 75.0 |  |  | 75.0 |
|  | Recall score | 90.9 | 77.8 |  | 90.9 | 55.6 |  | 81.8 | 66.7 |  | 81.8 | 66.7 |  |
|  | Precision score | 83.3 | 87.5 |  | 71.4 | 83.3 |  | 75.0 | 75.0 |  | 75.0 | 75.0 |  |
|  | F1/ F2 scores | 86.9/ 89.2 | 82.3/ 79.5 |  | 80.0/ 86.2 | 66.7/ 59.5 |  | 78.2/ 80.3 | 70.58/ 68.1 |  | 78.2/ 80.3 | 70.5/ 68.1 |  |
| Random Forest | Mean accuracy |  |  | 75.0 |  |  | 55.0 |  |  | 55.0 |  |  | 60.0 |
|  | Recall score | 90.9 | 55.6 |  | 100.0 | 0.0 |  | 100.0 | 0.0 |  | 90.9 | 22.2 |  |
|  | Precision score | 71.4 | 83.3 |  | 55.0 | 0.0 |  | 55.0 | 0.0 |  | 58.8 | 66.7 |  |
|  | F1/ F2 scores | 80.0/86.2 | 66.7/59.5 |  | 70.9/ 85.9 | 0.0/ 0.0 |  | 70.9/ 85.9 | 0.0/ 0.0 |  | 71.4/ 81.9 | 33.3/ 25.6 |  |
| Adaboost | Mean accuracy |  |  | 80.0 |  |  | 65.0 |  |  | 80.0 |  |  | 65.0 |
|  | Recall score | 90.9 | 66.7 |  | 81.8 | 44.4 |  | 100.0 | 55.6 |  | 90.9 | 33.3 |  |
|  | Precision score | 76.9 | 85.7 |  | 64.2 | 66.7 |  | 73.3 | 100.0 |  | 62.5 | 75.0 |  |
|  | F1/ F2 scores | 83.3/ 87.7 | 75.0/ 69.7 |  | 72.0/ 77.5 | 53.3/ 47.6 |  | 84.6/ 93.2 | 71.4/ 60.9 |  | 74.0/ 83.3 | 46.1/ 37.5 |  |
| Decision Tree | Mean accuracy |  |  | 85.0 |  |  | 60.0 |  |  | 45.0 |  |  | 60.0 |
|  | Recall score | 90.9 | 77.8 |  | 81.8 | 33.3 |  | 54.5 | 33.3 |  | 90.9 | 22.2 |  |
|  | Precision score | 83.3 | 87.5 |  | 60.0 | 60.0 |  | 50.0 | 37.5 |  | 58.8 | 66.7 |  |
|  | F1/ F2 scores | 86.9/ 89.2 | 82.3/79.5 |  | 69.2/ 76.2 | 42.8/ 36.5 |  | 52.1/ 53.5 | 35.2/ 34.0 |  | 71.4/ 81.9 | 33.3/ 25.6 |  |
| k- nearest neighbor | Mean accuracy |  |  | 70.0 |  |  | 55.0 |  |  | 75.0 |  |  | 75.0 |
|  | Recall score | 90.9 | 44.4 |  | 81.8 | 22.2 |  | 100.0 | 44.4 |  | 90.9 | 55.6 |  |
|  | Precision score | 66.7 | 80.0 |  | 56.2 | 50.0 |  | 68.7 | 100.0 |  | 71.4 | 83.3 |  |
|  | F1/ F2 scores | 76.9/ 84.7 | 57.1/ 48.7 |  | 66.7/ 75.0 | 30.7/ 25.0 |  | 81.4/ 91.6 | 61.5/ 50.0 |  | 80.0/86.2 | 66.7/59.5 |  |

ASD = Autism spectrum disorder; TD = Typically developing; Schi = Schizophrenia; All = Whole brain or all features combined.
